# DJ-1-Dependent Regulation of Oxidative Stress in the Retinal Pigment Epithelium (RPE)

**DOI:** 10.1371/journal.pone.0067983

**Published:** 2013-07-02

**Authors:** Karen G. Shadrach, Mary E. Rayborn, Joe G. Hollyfield, Vera L. Bonilha

**Affiliations:** Department of Ophthalmic Research, The Cole Eye Institute, Cleveland Clinic Lerner College of Medicine, Cleveland, Ohio, United States of America; University of Kentucky, United States of America

## Abstract

**Background:**

DJ-1 is found in many tissues, including the brain, where it has been extensively studied due to its association with Parkinson’s disease. DJ-1 functions as a redox-sensitive molecular chaperone and transcription regulator that robustly protects cells from oxidative stress.

**Methodology:**

Retinal pigment epithelial (RPE) cultures were treated with H_2_O_2_ for various times followed by biochemical and immunohistological analysis. Cells were transfected with adenoviruses carrying the full-length human DJ-1 cDNA and a mutant construct, which has the cysteine residues at amino acid 46, 53 and 106 mutated to serine (C to S) prior to stress experiments. DJ-1 localization, levels of expression and reactive oxygen species (ROS) generation were also analyzed in cells expressing exogenous DJ-1 under baseline and oxidative stress conditions. The presence of DJ-1 and oxidized DJ-1 was evaluated in human RPE total lysates. The distribution of DJ-1 was assessed in AMD and non-AMD cryosectionss and in isolated human Bruch’s membrane (BM)/choroid from AMD eyes.

**Principal Findings:**

DJ-1 in RPE cells under baseline conditions, displays a diffuse cytoplasmic and nuclear staining. After oxidative challenge, more DJ-1 was associated with mitochondria. Increasing concentrations of H_2_O_2_ resulted in a dose-dependent increase in DJ-1. Overexpression of DJ-1 but not the C to S mutant prior to exposure to oxidative stress led to significant decrease in the generation of ROS. DJ-1 and oxDJ-1 intensity of immunoreactivity was significantly higher in the RPE lysates from AMD eyes. More DJ-1 was localized to RPE cells from AMD donors with geographic atrophy and DJ-1 was also present in isolated human BM/choroid from AMD eyes.

**Conclusions/Significance:**

DJ-1 regulates RPE responses to oxidative stress. Most importantly, increased DJ-1 expression prior to oxidative stress leads to decreased generation of ROS, which will be relevant for future studies of AMD since oxidative stress is a known factor affecting this disease.

## Introduction

The retinal pigment epithelium (RPE) constitutes a monolayer of cuboidal cells. Its apical surface faces a very complex extracellular matrix called the interphotoreceptor matrix (IPM) that also surrounds the photoreceptor cells projecting from the outer retina. The RPE basal surface faces the underlying Bruch’s membrane (BM) [1]. The RPE exhibit a number of highly specialized functions, including phagocytosis of shed tips of photoreceptor outer segments, directional transport of nutrients into and removal of waste products from photoreceptor cells, optimization of ion concentrations in the surrounding tissues, elimination of fluid from the IPM, and visual pigment regeneration and transport which, is essential to retinal homeostasis and vision.

The RPE cells live under chronic oxidative stress because of the lifelong exposure to light, high oxygen consumption and high oxygen partial pressure from the underlying choriocapillaris. In addition, phagocytosed shed photoreceptor outer segments are enriched in polyunsaturated fatty acids such as docosahexaenoate (DHA) and retinoids, which are highly susceptible to lipid peroxidation and fracgmentation leading to lipid peroxide–derived protein modifications [2], [3]. Finally, phagocytosis itself increases oxidative stress resulting in the generation of endogenous reactive oxygen species (ROS) [4], [5]. This hostile oxidative environment is thought to contribute to retinal disease. Indeed, it was suggested that oxidative stress affecting the physiological function and leading to focal loss of the RPE cells is a major factor contributing to geographic atrophy, and vision loss in the elderly blinding disease age-related macular degeneration (AMD) [6], [7]. However, it remains to be determined why the initial retinal degeneration occurs and how degeneration processes progress as a result of continued oxidative insults.

Deletion or homozygous mutations of DJ-1 gene (*PARK7* locus) have been shown to cause early-onset autosomal recessive Parkinson’s disease (PD) [8]. The DJ-1 gene encodes a highly conserved protein with 189 amino acids and a molecular weight of ∼20 kDa. DJ-1 has been implicated in diverse cellular processes, including cellular transformation and tumorigenesis [9], [10], [11], transcriptional regulation [12], [13], [14], [15], androgen receptor signaling [16], chaperone [17], [18], spermatogenesis [19], and oxidative stress response [20], [21], [22].

Oxidative stress occurs when there is an imbalance between the biological processes that produce ROS and the protective mechanisms within cells that scavenge ROS. Several studies have demonstrated that DJ-1 robustly protects cells from oxidative stress through distinct cellular pathways [20], [21], [23], [24], [25], [26], [27], [28], [29]. DJ-1 can eliminate H_2_O_2_ by becoming oxidized itself and thus functioning as a scavenger of ROS [20], [21], [30], [31]. It was also proposed that some of the protective actions of DJ-1 might occur at the transcriptional level [32]. DJ-1 binds to PIAS proteins, a family of SUMO-1 ligases that modulate the activity of various transcription factors [16]. Wild-type DJ-1 sequesters the death protein Daxx in the nucleus, preventing it from binding and activating its effector kinase, apoptosis signal-regulating kinase 1 (Ask1) in the cytoplasm [33]. Others showed that DJ-1 is a transcriptional co-activator that interacts with the nuclear proteins p54nrb and PSF [34] again to protect against apoptosis. DJ-1 also stabilizes the antioxidant transcriptional master regulator nuclear factor erythroid-2 related factor 2 (Nrf2) [15], [35] by preventing association with its inhibitor protein Keap1. Therefore, DJ-1 may act as a transcriptional co-factor that regulates the response to oxidative stress. DJ-1 has also been reported to confer protection against endoplasmic reticulum (ER) stress, proteasomal inhibition, and toxicity induced by overexpression of Pael-R [36]. Recently reported data showed that the apparently pleiotropic roles of DJ-1 seem to be related to the single function of binding multiple mRNA transcripts with a GG/CC-rich sequence [37]. More recently it was shown that DJ-1 plays a role in maintenance of mitochondria structure by counteracting the mitochondrial impairment induced by the tumour suppressor protein p53 [38]. Moreover, overexpression of the gene encoding DJ-1 protects against oxidative injury whereas knocking down the expression by RNAi enhances susceptibility to oxidative stress [21], [36], [39], [40], [41], [42], [43]. Thus, DJ-1 may play a crucial role both sensing and conferring protection against a range of oxidative stressors, through multiple mechanisms.

Previously, we have identified DJ-1 peptides in both young and aged RPE lysates using a proteomic approach [44]. In this study, we describe DJ-1 expression, distribution and function in RPE cells under baseline conditions and following oxidative stress. We also analyze DJ-1 and oxDJ-1 levels in human RPE lysates from non-AMD and AMD donors. Finally, we describe DJ-1 distribution in the RPE from non-AMD and AMD donors with geographic atrophy, and in isolated human BM/choroid (with drusen) from AMD eyes. Adaptation to changes in oxidative environments is critical for the survival of retina and the RPE. Therefore, knowledge of the DJ-1 function in oxidative stress in the RPE will provide insight into biochemical processes that support and maintain vision in physiological and pathological conditions.

## Results

### DJ-1 Expression and Distribution in RPE Cell Cultures

**Figure 1:**
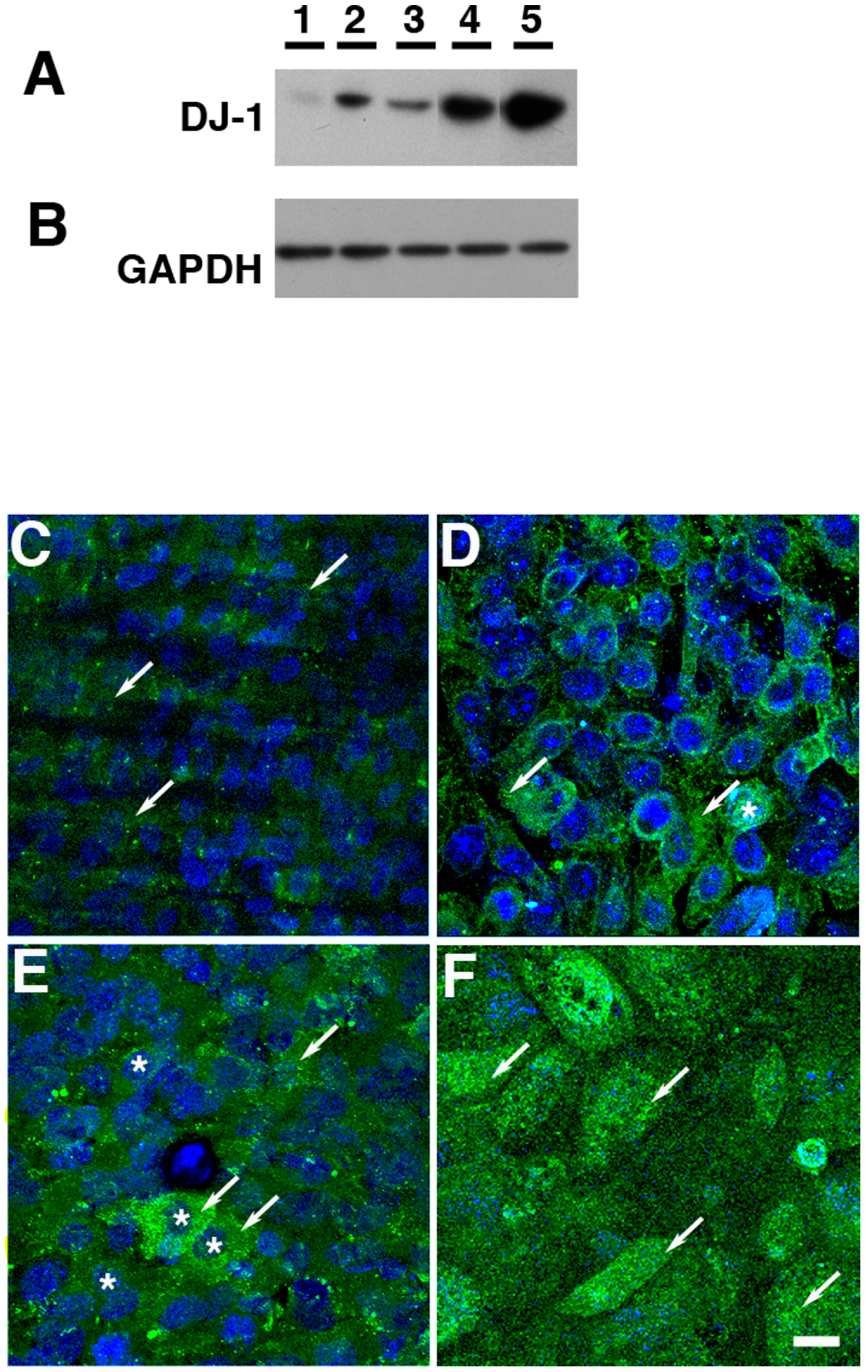
DJ-1 is present in RPE cell cultures. Lysates of the human RPE cell lines ARPE-19 (A, lane 1) and D407 (A, lane 2), the mouse RPE cell line B6-RPE07 (A, lane 3), mouse primary RPE (A, lane 4) and mouse brain lysates (A, lane 5) were harvested and analyzed by immunoblot assay with DJ-1 antibody. The DJ-1 signal varied in intensity in each RPE cell culture. Each lane contained 20 µg of protein. Protein loads were confirmed in replicate blots probed with GAPDH (B). Alternatively, monolayers of ARPE-19 (C), D407 (D), B6-RPE07 (E) and mouse primary RPE (F) were fixed with paraformaldehyde before extraction with Triton X-100 and labeling with DJ-1 antibodies. Cell nuclei were labeled with TO-PRO-3. DJ-1 labeling was diffusely distributed in the cytoplasm (arrows) and in the nuclei (*) of some of the cells (C-F, arrows). Scale bar = 20 µm.

Recently, we identified DJ-1 peptides in both young and aged RPE lysates using proteomic analysis [44]. To decipher the molecular mechanism of DJ-1 function we decided to use several previously characterized RPE cell cultures. One was the newly characterized B6-RPE07 mouse RPE cell line, capable of displaying a polarized phenotype when cultured in a serum free epithelial medium and plated on collagen-coated Transwells [45]. We also used two human RPE cell lines: ARPE-19 and D407. We also used mouse primary RPE cultures, which morphologically resemble RPE cells *in* *vivo* due to their elaboration of apical microvilli and basal infoldings on their surfaces [46]. The initial characterization of these four cell lines analyzed the expression of DJ-1 by Western analysis (Fig. 1A and B) and immunofluorescence (Fig. 1C to F). A major band of ∼25 kDa was observed in the extracts of all the RPE cell lines (Fig. 1A, lanes 1 to 3) and mouse primary RPE (Fig. 1A, lane 5) when compared to extracts from mouse brain (Fig. 1A, lane 4). Immunoblots of RPE lysates obtained from all cell cultures demonstrated heterogeneity in the levels of expression of DJ-1 when compared to the expression of the loading control protein GAPDH (Fig. 1B). DJ-1 expression in mouse primary RPE lysates displayed the highest levels of expression of DJ-1 among the RPE cells tested (Fig. 1A, lane 5). Confocal microscopy *en face* examination of paraformaldehyde-fixed monolayers grown on polycarbonate filters revealed that under baseline conditions, DJ-1 displays a diffuse cytoplasmic and, in some cells, nuclear staining (Fig. 1C to F, arrows) in all the RPE cell lines analyzed. DJ-1 analysis in polarized RPE monolayers established that although each one of the RPE cell cultures have different levels of DJ-1, they all display similar subcellular distribution of DJ-1.

### DJ-1 Expression in RPE Cells Subjected to Oxidative Stress

**Figure 2:**
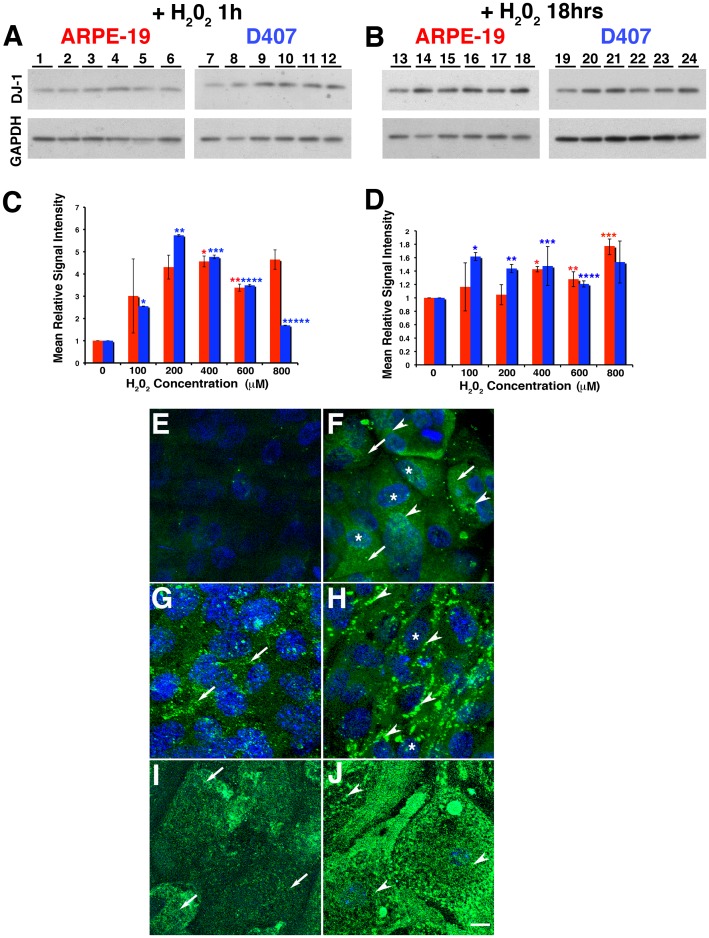
Oxidative stress induced by H_2_O_2_ increases DJ-1 levels and leads to intracellular redistribution of DJ-1 in RPE cells. ARPE-19 and D407 monolayers were treated with increasing concentrations (0 to 800 µM) of H_2_O_2_ for 1 hr (A) and 18 hrs (B), harvested, and analyzed by immunoblot assay with DJ-1 antibody (upper panel). Each lane contained 20 µg of protein. Protein loadings were confirmed in replicate blots probed with GAPDH (lower panel). A representative Western is shown. A dose response of ARPE-19 (A, lanes 1 to 6) and D407 (A, lanes 7 to 12) is observed when cells are exposed to increasing concentrations of H_2_O_2_ for 1 hr. Quantitation of these blots showed that DJ-1 immunoreactivity was 5.0 and 3.6 fold higher in ARPE-19 incubated with 400 and 600 µM H_2_O_2_ and up to 5.7 fold higher in D407 cells incubated with 200 µM H_2_O_2_ when compared with control cell RPE cultures (C). Plotted signals represent the intensity for each band normalized to GAPDH signal and compared to the intensity of the control, untreated cells (lanes 1, 7, 13, 19). Red columns = ARPE-19; blue columns = D407 cells. Data is expressed as mean relative signal intensity ± SEM (n = 3). Asterisks denote statistical significance compared with control untreated cells (*p = 0.0160 and **p = 0.0145 in the ARPE-19 and *p<0.0001, **p<0.0001, ***p = 0.0005, ****p = 0.0004 and p***** = 0.0001 in D407 cells). Similarly, both ARPE-19 (Fig. B, lanes 13 to 18) and D407 (Fig. 2b, lanes 19 to 24) also displayed a dose response when cells were exposed to increasing concentrations of H_2_O_2_ for 18 hrs. Quantitation of these blots showed that DJ-1 immunoreactivity was 1.4, 1,3 and 1.8 fold higher in ARPE-19 incubated with 400 to 800 mM H_2_O_2_ and up to 1.6 fold higher in D407 cells incubated with 100 to 800 µM H_2_O_2_ when compared with control cell RPE cultures (D). Asterisks denote statistical significance compared with control untreated cells (*p = 0.0010, **p = 0.0146 and ***p = 0.0185 in the ARPE-19 and *p = 0.0005, **p = 0.0020, ***p = 0.0177 and ****p = 0.0103 in D407 cells). **E–J.** Confocal immunofluorescence staining of ARPE-19 (E, F), B6-RPE07 (G, H) and mouse primary RPE cultures (I, J) fixed before extraction with Triton X-100 and labeling with DJ-1 antibody. Cell nuclei were labeled with TO-PRO-3. Observations demonstrated that at baseline conditions, DJ-1 is diffused in the cytoplasm (arrows) and nuclei (*) of polarized RPE cells (E, G, I). With 18 hrs of exposure to 400 µM H_2_O_2_ (F, H, J), the diffused cytoplasmic DJ-1 staining disappears and pronounced aggregated perinucler staining (arrowheads) for DJ-1 is apparent. Scale bar = 10 µm.

To evaluate the role of oxidative stress on DJ-1 expression, RPE cultures were plated, incubated with H_2_O_2_ and the expression and distribution of DJ-1 was analyzed (Fig. 2). A representative Western is shown. A dose response relating DJ-1 expression in ARPE-19 (Fig. 2A, lanes 1 to 6) and D407 (Fig. 2A, lanes 7 to 12) is observed when cells are exposed to increasing concentrations of H_2_O_2_ for 1 hr. While exposure of ARPE-19 cells to H_2_O_2_ at 400 µM was sufficient to significantly enhance DJ-1 levels, a lower concentration (100 µM) was adequate to modulate DJ-1 levels in D407 cells. Quantitation of the intensity of immunoreactivity in blots from three independent experiments showed that DJ-1 increased 5.0 and 3.6 fold in ARPE-19 incubated with 400 and 600 µM H_2_O_2_ and up to 5.7 fold in D407 cells incubated with 200 µM H_2_O_2_ when compared with control cell RPE cultures (Fig. 2C). Similarly, both ARPE-19 (Fig. 2B, lanes 13 to 18) and D407 (Fig. 2b, lanes 19 to 24) also displayed a dose response when cells were exposed to increasing concentrations of H_2_O_2_ for 18 hrs. Quantitation of these blots showed that the intensity of DJ-1 immunoreactivity was 1.4, 1.3 and 1.8 fold higher in ARPE-19 incubated with 400 to 800 µM H_2_O_2_ and up to 1.6 fold greater in D407 cells incubated with 100 to 800 mM H_2_O_2_ when compared with control cell RPE cultures (Fig. 2D). Finally, ARPE-19 and D407 monolayers exposed to oxidative stress induced by incubation with increasing concentration of 4-hydroxynonenal (4-HNE), a lipid peroxidation product, for 12 (Figure S1) and 24 hrs (data not shown) also displayed increased DJ-1 immunoreactivity.

We also analyzed the distribution of DJ-1 in several RPE cell cultures plated on Transwell inserts non-incubated or incubated with 100 µM H_2_O_2_ overnight followed by immunofluorescence (Fig. 2E to J). Confocal microscopy *en face* examination of paraformaldehyde-fixed monolayers revealed a diffuse staining for DJ-1 consistent with a cytoplasmic localization in ARPE-19 (Fig. 2E), B6-RPE07 (Fig. 2G) and in mouse primary RPE (Fig. 2I) monolayers under baseline conditions. Oxidative stress induced by H_2_O_2_ leads to a visible increase in immunocytochemical staining for DJ-1 (Fig. 2F, H, J). In addition, to this visible increase in DJ-1 staining, an induced intracellular redistribution of DJ-1 to an aggregated, perinuclear localization was also observed (Fig. 2F, H, J). Our results suggested that DJ-1 immunoreactivity is increased and redistributed within RPE cells subjected to oxidative stress.

### DJ-1 Localization in RPE Cells Subjected to Oxidative Stress

**Figure 3:**
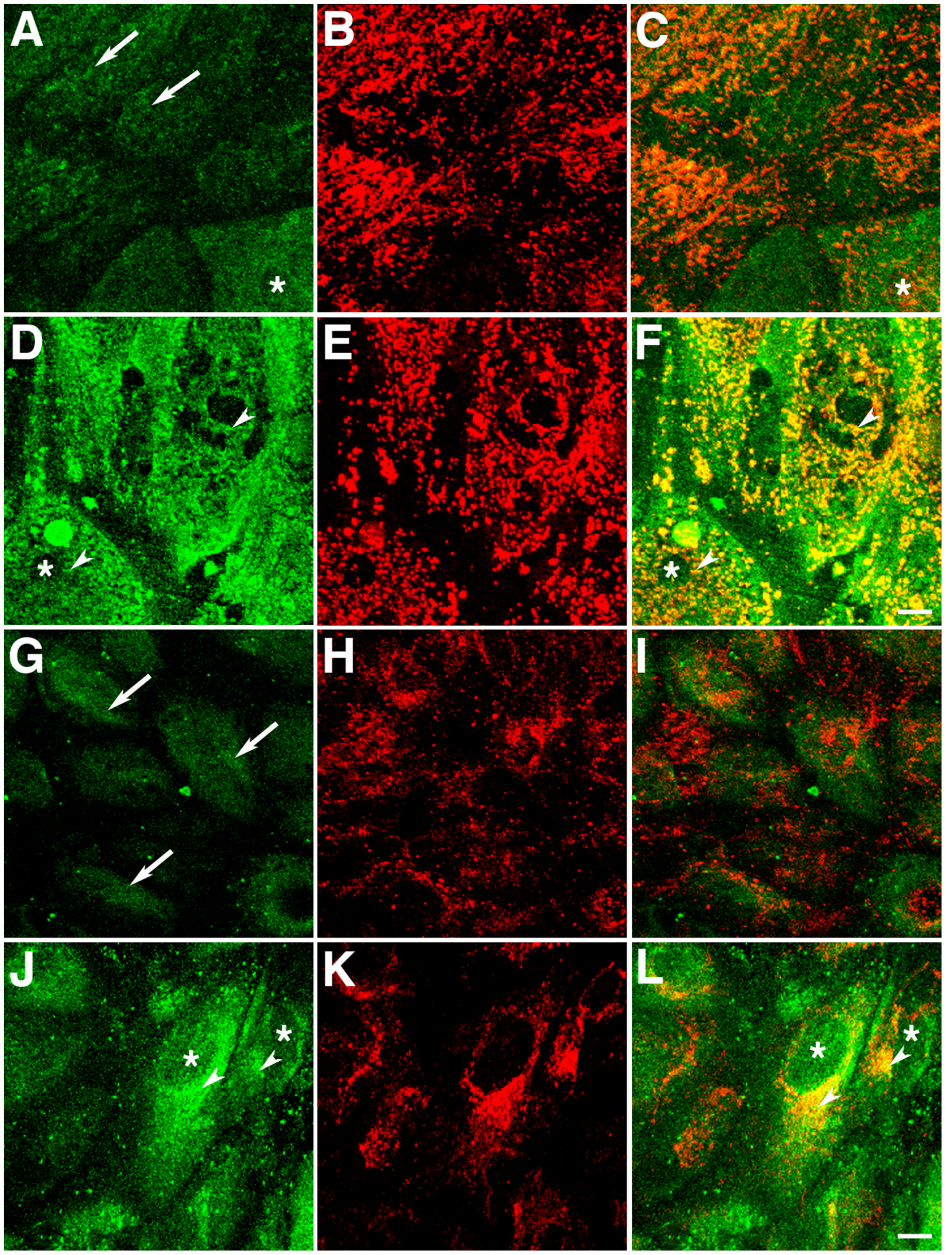
Oxidative stress-dependent translocation of DJ-1 into mitochondria. Representative confocal micrographs of mouse primary RPE (A–F) and ARPE-19 (G–L) monolayers plated on Transwells® and labeled with antibodies to DJ-1 (A, D, G, J) and COX IV (B, E, H, K). Under baseline conditions, there is very little colocalization between DJ-1 and COX IV, as observed in overlaid images (C, I) for both RPE cultures; DJ-1 is mostly distributed trough the cytoplasm (arrows) and to the nuclei (*) of some cells. Upon oxidative stress induced by incubation with 400 µM H_2_O_2_ for 1 hr, DJ-1 staining is increased both in the mouse primary (D) and ARPE-19 (J) cultures. In cultures treated with H_2_O_2_ some DJ-1 re-distributed to mitochondria (arrowheads) and displayed significant colocalization with COX IV in overlaid images (F, L). Scale bar = 10 µm.

Further experiments were carried out in several RPE cultures plated on Transwell inserts non-incubated or incubated with 100 µM H_2_O_2_ overnight followed by immunofluorescence to confirm the intracellular localization of DJ-1 upon oxidative injury (Fig. 3). Confocal microscopy *en face* examination of paraformaldehyde-fixed monolayers again revealed a diffuse staining for DJ-1 consistent with a cytoplasmic localization in mouse primary RPE (Fig. 3A) and in ARPE-19 (Fig. 2G) monolayers. Monolayers were also labeled with antibodies specific to the mitochondrial marker OxPhos Complex IV subunit I (COX IV, Fig. 3B, E, H, K). Under baseline conditions, both RPE monolayers displayed no significant colocalization with the mitochondrial marker in overlaid images (Fig. 3C and I). Oxidative stress induced by H_2_O_2_ lead to a visible increase in immunocytochemical staining for DJ-1 (Fig. 3D and J). In addition, an intracellular redistribution of DJ-1 immunoreactivity leading to COX IV colocalization was also observed (Fig. 3F and L).

Additional experiments carried out in RPE cells plated on coverslips and incubated with the mitochondrial staining MitoTracker were also performed (Figure S2). B6-RPE07 monolayers were fixed and labeled for DJ-1 (Figure S2A and S2D) after loading with the mitochondrial staining MitoTracker (Figure S2B and S2E). Similar to results reported above with antibody labeling, DJ-1 showed little specific localization to mitochondria under baseline conditions (Figure S2A and S2C). However, treatment with H_2_O_2_ caused increased redistribution of DJ-1 to mitochondria as shown in the overlain images (Figure S2D and S2F, arrows). In addition, an increase in mitochondria labeling is also evident in the RPE cells subjected to oxidative stress. Together, our observations indicate that DJ-1 is redistributed to the mitochondria in RPE cells under oxidative stress.

### Detection of DJ-1 Oxidation at Cysteine 106 (oxDJ-1) in Cells Subjected to Oxidative Stress

**Figure 4:**
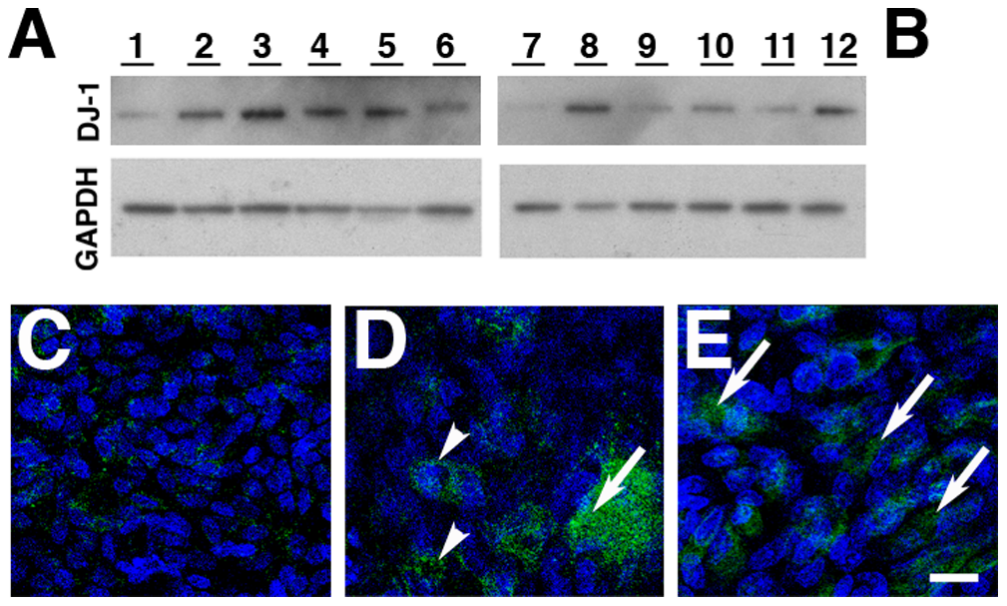
Presence of oxDJ-1 in RPE cells subjected to oxidative stress. ARPE-19 monolayers were treated with increasing concentrations (0 to 800 µM) of H_2_O_2_ for 1 hr (A) and 18 hs (B), harvested, and analyzed by immunoblot assay with oxDJ-1 antibody (upper panel). Protein loadings were confirmed in replicate blots probed with GAPDH (lower panel). Each lane contained 20 µg of protein. A dose response is observed when cells are exposed to increasing concentrations of H_2_O_2_ for 1 h (A, lanes 1 to 6) and 18 hrs (B, lanes 7 to 12). Confocal immunofluorescence staining of baseline ARPE-19 cultures (C) fixed before extraction with Triton X-100 and labeling with oxDJ-1 antibodies revealed absence of oxDJ-1. However, oxDJ-1 is observed in the cytoplasm (arrows) and perinuclear area (arrowheads) of RPE cells exposure to 400 µM H_2_O_2_ for 1 h (D) and 18 hrs (E). Cell nuclei were labeled with TO-PRO-3. Scale bar = 20 µm.

DJ-1 has three cysteine (C) residues at amino acids 46, 53 and 106. C106 in DJ-1 is the first to become oxidized by the addition of cysteine sulfinic acid (C-SO_2_H) and then C46 and C53 become oxidized upon oxidative stress, resulting in scavenging of reactive oxidative species (ROS) and enhancing DJ-1 association with mitochondria [23], [47], [48]. To check for alterations in DJ-1 oxidative stress, ARPE-19 cultures at baseline and under oxidative stress conditions were reacted with an antibody generated against a synthetic peptide containing SO_3_H at C106 of DJ-1 [49]. If RPE cells under stress generate DJ-1 oxidized at C106, it may be increased during this time. Immunoblots of lysates of ARPE-19 cells obtained from cultures subjected to oxidative stress induced by exposure to H_2_O_2_ for 1 hr (Fig. 4A) and 18 hrs (Fig. 4B) demonstrated a progressive increase in oxDJ-1 with a dose response increase in cultures under short-term oxidative tress (Fig. 4A). Interestingly, the oxDJ-1 detected mostly in cultures under oxidative stress, displayed a molecular weight ∼110 kDa. We also tested ARPE-19 cultures for the expression of oxDJ-1 by immunofluorescence (Fig. 4C to E). Polarized monolayers plated on Transwells under baseline culture conditions failed to display labeling with the antibody to oxDJ-1 (Fig. 4C). However, several cells displayed staining with the antibody to oxDJ-1 when monolayers were incubated with H_2_O_2_ for 1 h (Fig. 4D) and 18 hrs (Fig. 4E). The large increase of oxDJ-1 content when RPE cultures were under oxidative stress suggests that this DJ-1 in RPE indeed undergoes oxidation under oxidative stress.

### Regulation and Distribution of DJ-1 Oxidation at C Residues in Cells Subjected to Oxidative Stress

**Figure 5:**
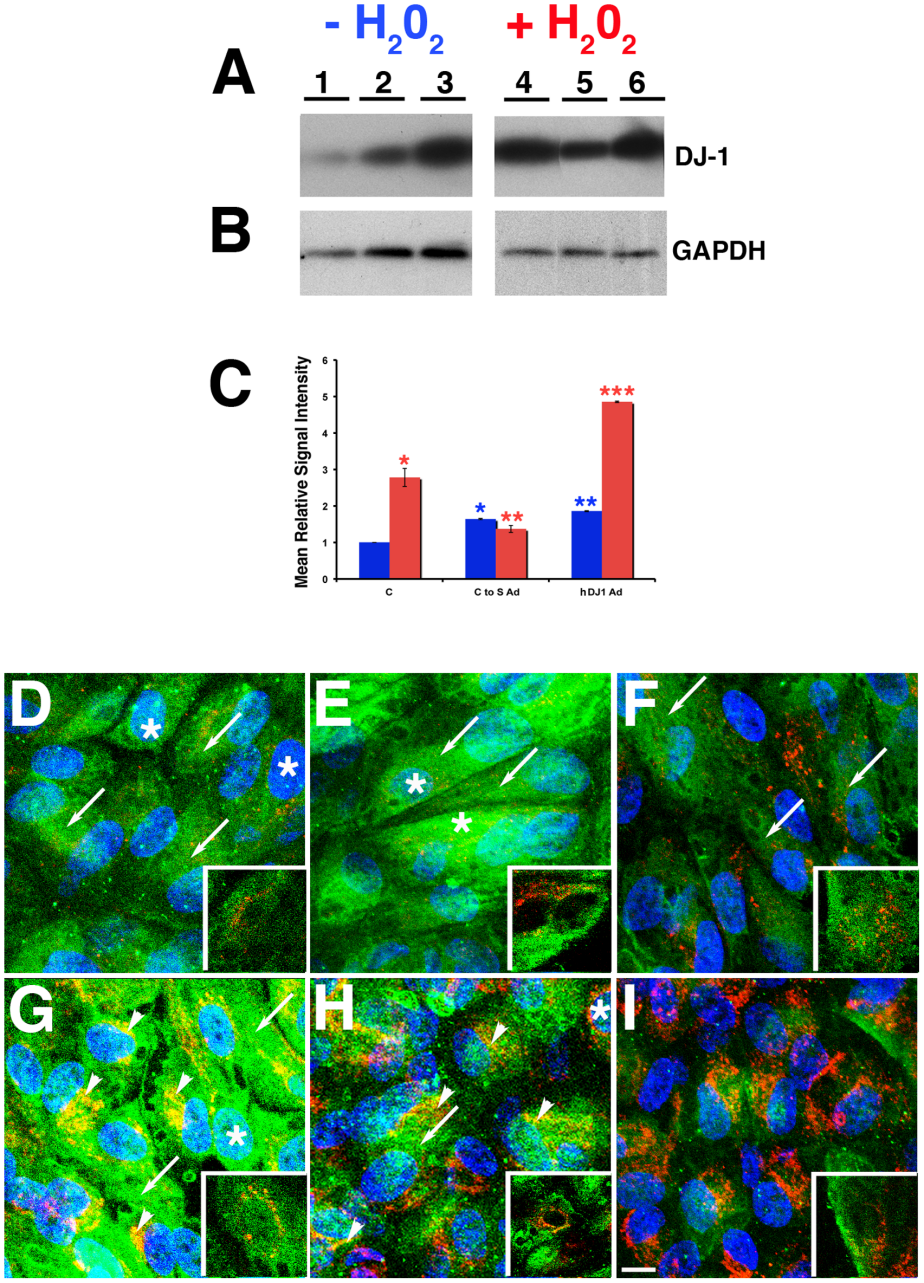
Overexpression of DJ-1 full-length lead to increased levels and redistribution in RPE cells upon oxidative stress. ARPE-19 monolayers were transduced with high titer (5×10^6^ pfu) adenovirus carrying full length human DJ-1 (hDJ-1 Ad) or adenovirus carrying DJ-1 C to S mutant (C to S Ad). Forty-eight hours later cultures were treated (A, lanes 4 to 6) or not (A, lanes 1 to 3) with 400 µM H_2_O_2_, harvested, and analyzed by immunoblot assay with DJ-1 antibody (A). Each lane contained 20 µg of protein. Protein loads were confirmed in replicate blots probed with GAPDH (B). Immunoblots of lysates revealed that ARPE-19 cultures transduced with the hDJ-1 (A, lane 3) and with the C to S Ad constructs (A, lane 2) displayed significant increased immunoreactivity of DJ-1 when compared to ARPE-19 control cultures (A, lane 1) under baseline conditions. Quantification of immunoblots of these cultures demonstrated a 1.6 and 1.8 fold increase in the expression levels of DJ-1 in ARPE-19 transduced with the C to S and hDJ-1 Ad, respectively (C, blue columns). Blue columns = baseline ARPE-19 cultures; Red columns = ARPE-19 incubated with 400 µM H_2_O_2_ for 1 hr. Data expressed as mean relative signal intensity ± SEM (n = 3). Asterisks denote statistical significance compared with control ARPE-19 untreated cells (*p = 0.0007 in ARPE-19 transduced with C to S Ad and **p<0.0001 ARPE-19 transduced with hDJ-1 Ad). In addition, a significant increase in the immunoreactivity of DJ-1 was observed when ARPE-19 cultures (A, lane 4) and ARPE-19 overexpressing the hDJ-1 Ad were subjected to oxidative stress (A, lane 6). Quantification of the immunoblots of these lysates demonstrated a 2.8, 1.4 and 4.8 fold increase in the immunoreactivity of DJ-1 in ARPE-19 cells and in ARPE-19 cells transduced with the C to S and hDJ-1 Ad, respectively (C, red columns), when comparing signal intensities to the one of ARPE-19 not overexpressing exogenous DJ-1 in baseline culture conditions. These differences were statistically significant (*p = 0.0020 in the ARPE-19, **p = 0.0177 in ARPE-19 transduced with C to S Ad and ***p<0.0001 ARPE-19 transduced with hDJ-1 Ad). **D–I:** Confocal microscopy of ARPE-19 monolayers fixed, permeabilized with a buffer containing Triton X-100, and processed for indirect immunofluorescence with DJ-1 (green) and COX IV (red) antibodies. Cell nuclei were labeled with TO-PRO-3. Under baseline conditions there is very little colocalization between DJ-1 and COX IV in control ARPE-19 (D) and in ARPE-19 transduced with hDJ-1 Ad (E) and C to S Ad (F); DJ-1 is distributed in the cytoplasm (arrows) and nuclei (*) of cells. Upon oxidative stress induced by incubation with 400 µM H_2_O_2_ for 1 h, DJ-1 staining redistributed intracellularly to mitochondria (arrowheads) and colocalized with COX IV both in ARPE-19 (G) and ARPE-19 transduced with hDJ-1 Ad (H) but not in ARPE-19 cultures transduced with the C to S Ad (I). Insets: close up images of the DJ-1 and COX IV labeling. Scale bar = 10 µm.

The above data suggested that oxidation of DJ-1 C residues is correlated to the presence of oxidative stress. To further understand the function of DJ-1 oxidation RPE cultures were infected with adenoviruses carrying full-length human DJ-1 cDNA (hDJ-1 Ad) and a mutant construct, which has the C residues at amino acids 46, 53 and 106 mutated to serine (S), (C to S Ad) prior to stress experiments followed by evaluation of content and localization of DJ-1 in RPE cultures (Fig. 5). Immunoblots of lysates revealed that ARPE-19 cultures transduced with the hDJ-1 (Fig. 5A, lane 3) and with the C to S Ad constructs (Fig. 5, lane 2) displayed significant increased immunoreactivity of DJ-1 when compared to ARPE-19 control cultures (Fig. 5A, lane 1) and normalized to the levels of GAPDH (Fig. 5B) under baseline conditions. Quantification of these lysates demonstrated a 1.6 and 1.8 fold increase in the content of DJ-1 in ARPE-19 transduced with the C to S and hDJ-1 Ad, respectively (Fig. 5C), when comparing DJ-1 immunoreactivity to the one of ARPE-19 with normal levels of DJ-1. In addition, a significant increase in the immunoreactivity of DJ-1 was observed when ARPE-19 cultures were subjected to oxidative stress induced by H_2_O_2_ (Fig. 5A, +H_2_O_2_). Quantification of the immunoblots of these lysates demonstrated a 2.8, 1.4 and 4.8 fold increase in the immunoreactivity of DJ-1 in ARPE-19 cells and in ARPE-19 cells transduced with the C to S and hDJ-1 Ad, respectively (Fig. 5C), when comparing signal intensities to the one of ARPE-19 DJ-1 levels in baseline culture conditions.

To compare the distribution of endogenous and exogenous DJ-1 under baseline and oxidative stress conditions, ARPE-19 cells and ARPE-19 cells transducing exogenous DJ-1 were fixed and labeled for the distribution of DJ-1 and mitochondria (Fig. 5D to I). Double labeling cells on Transwells with antibodies to DJ-1 (green) and the mitochondria protein COX IV (red) showed little colocalization of DJ-1 with COX IV in control ARPE-19 cultures (Fig. 5D) and in ARPE-19 overexpressing full-length DJ-1 (Fig. 5E) and the C to S mutant DJ-1 (Fig. 5F) under baseline culture conditions. However, a significant colocalization of DJ-1 with COX IV could be observed in ARPE-19 cells (Fig. 5G and inset) and ARPE-19 cultures overexpressing full-length DJ-1 (Fig. 5H and inset) when incubated with H_2_O_2_. Opposing, ARPE-19 cultures overexpressing the C to S mutant DJ displayed no colocalization between the distribution of DJ-1 and COX IV (Figure 5I and inset) under oxidative stress. Altogether, our results showed that DJ-1 C residues are important for their increased response and redistribution to the mitochondria in cells subjected to oxidative stress.

### Functional Role of DJ-1 C Oxidation in Cells Subjected to Oxidative Stress

**Figure 6:**
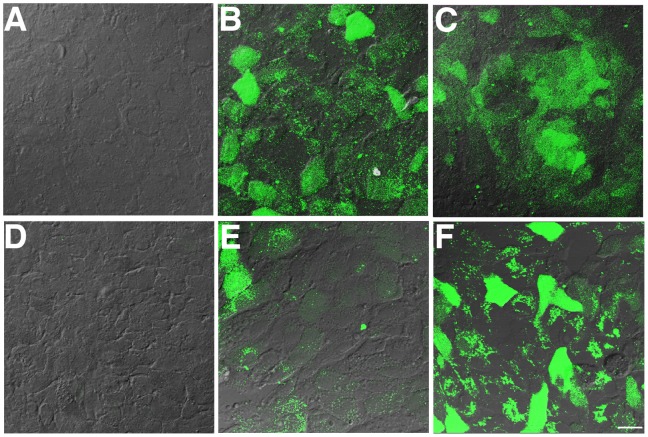
Overexpression of DJ-1 full length prior to oxidative stress leads to decreased levels of H_2_O_2_-induced ROS generation by RPE cells. ARPE-19 control cultures (A, B) and ARPE-19 transduced with adenovirus carrying full length human DJ-1 (hDJ-1 Ad) or adenovirus carrying DJ-1 C to S mutant (C to S Ad). Forty-eight hours following infection with adenovirus, cultures were treated with 800 µM H_2_O_2_ for 1 h followed by incubation with CM-H_2_DCFDA and the fluorescence intensity of oxidized DCF (green) was then visualized by confocal microscopy. Cellular images were obtained by difference interference contrast (DIC) and overlaid on the fluorescence image of generated ROS. Images indicated that no ROS generation is observed in RPE cells at basal conditions (A) while upon oxidative stress incubation, appreciable ROS generation throughout the entire cell body of cells is observed (B). RPE cultures transduced with the high titer (5×10^6^ pfu) of the C to S Ad displayed appreciable ROS generation when cells were exposed to oxidative stress (C). Markedly, RPE cultures transduced with the high titer of the hDJ-1 Ad (5×10^6^ pfu) did not display ROS generation when exposed to oxidative stress (D). However, ROS generation was increasingly observed when RPE cultures were infected with increasing dilutions of hDJ-1 Ad, such as 4×10^6^ pfu (E) and 3×10^6^ pfu (F). Scale bar = 20 µm.

To determine whether DJ-1 oxidation at its C residues could protect RPE cells against oxidative stress, we subjected ARPE-19 cells and ARPE-19 cells overexpressing full-length and C to S mutant DJ-1 to oxidative stress induced by H_2_O_2_ and labeling of ROS generation through to incubation with CM-H_2_DCFDA (Fig. 6). No ROS were observed in ARPE-19 cells under normal culture conditions (Fig. 6A). However, significant intracellular ROS generation was observed when ARPE-19 (Fig. 6B) and ARPE-19 monolayers overexpressing the C to S mutant DJ-1 (Fig. 6C) were subjected to oxidative stress. Strikingly, no ROS was generated when ARPE-19 monolayers were overexpressing the full length DJ-1 (Fig. 6D). An inverse dose response of ARPE-19 monolayers overexpressing full length DJ-1 was observed when cells were infected with decreasing concentrations of hDJ-1 adenovirus (Fig. 6E and F), suggesting a gene-dosage effect. These results suggested that DJ-1 C oxidation is necessary for DJ-1 to protect RPE cells under oxidative stress from the intracellular generation of ROS.

### Detection of DJ-1 in Human RPE Cells Isolated from the Eyes from AMD and non-AMD Donors

**Figure 7:**
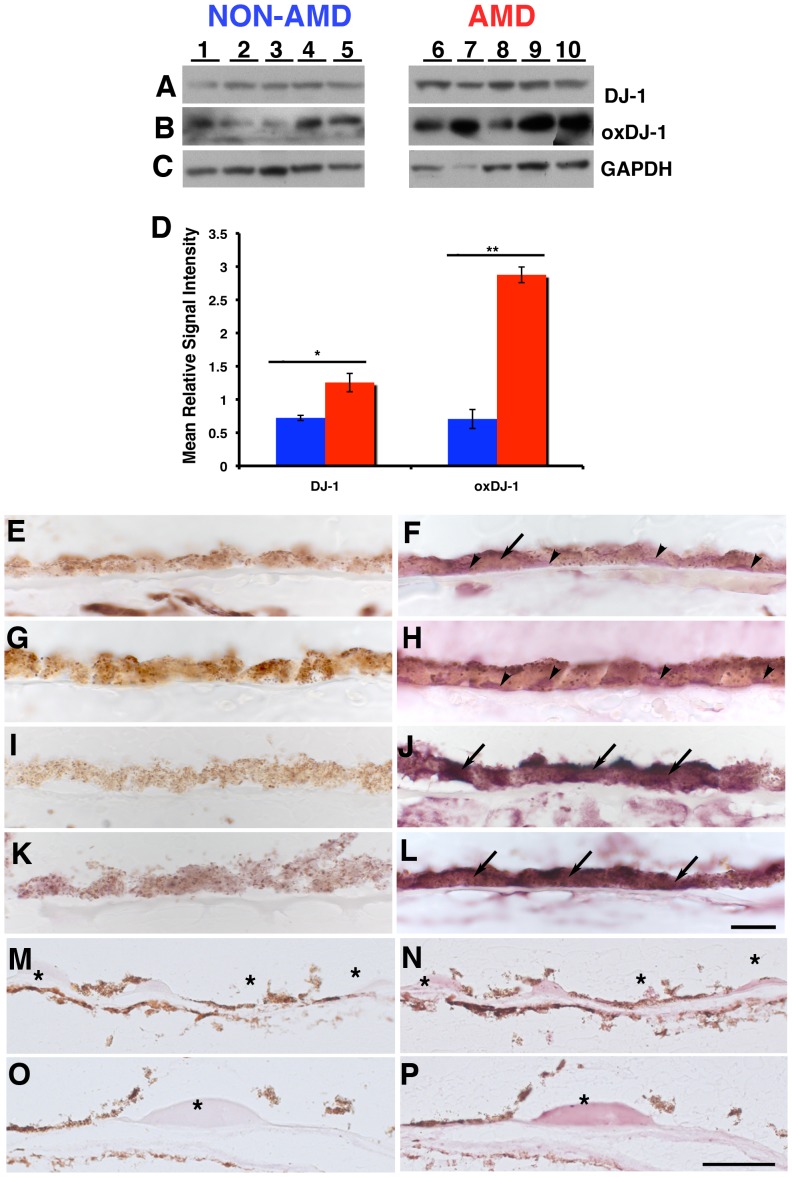
Increased levels of DJ-1 and oxDJ-1 in RPE lysates and tissue from AMD donors. Lysates of human RPE isolated from non-AMD (A to C lanes 1 to 5) and AMD donors (A to C, lanes 6 to 10) were harvested and analyzed by immunoblot assay with DJ-1 antibody (A) and oxDJ-1 antibody (B). Each lane contained 20 µg of protein. Protein loads were confirmed in replicate blots probed with GAPDH (C). Immunoblots of lysates revealed that AMD RPE displayed significant increased immunoreactivity of both DJ-1 (A) and oxDJ-1 (B) when compared to non-AMD RPE lysates. Quantification of immunoblots demonstrated a 1.7 and 4 fold increase in the expression levels of DJ-1 and oxDJ-1, respectively (D). Blue columns = non-AMD; Red columns = AMD. Data is expressed as mean relative signal intensity ± SEM (n = 8). Asterisks denote statistical significance compared with non-AMD RPE (*p = p = 0.0098 for DJ-1 and **p = 0.0058 for oxDJ-1). Alternatively, cryosections of different non-AMD (E–H) and AMD (I to L) donors with geographic atrophy, and isolated Bruch’s membrane (BM) and choroid from two different AMD donors were (M to P) labeled with DJ-1 antibody. Negative control sections were reacted with DJ-1 antibody pre-absorbed with lysates of cells overexpressing DJ-1 and shoed no DJ-1 labeling (E, G, I, K, M, O). DJ-1 labeling was detected mostly in the RPE nuclei (arrowheads) but also in the cytoplasm of non-AMD donors (F and H, arrows). Significantly more DJ-1 was detected all over the cytoplasm of RPE cells from two different AMD donors (J and L, arrows), while DJ-1 was diffusely distributed in isolated BM and in drusen (E, G, asterisks). Scale bars (E to L) = 10 µm; (M to P) = 50 µm.

To better understand the significance of our RPE culture findings, DJ-1 presence in RPE lysates isolated from human donor eyes was carried out. DJ-1 presence was also probed in RPE cells isolated from donors with AMD, a blinding disease affecting the elderly, with an established contribution of oxidative stress to the pathogenesis of the disease (Fig. 7). DJ-1 was detected and highly expressed in the RPE lysates from AMD donors (Fig. 7A, lanes 6 to 10) when compared to the RPE lysates from non-AMD donors (Fig. 7A, lanes 1 to 5). DJ-1 immunoreactivity was normalized to the GAPDH content of the samples (Fig. 7C). Quantitation of these blots showed that DJ-1 immunoreactivity was increased ∼2.5 fold in RPE isolated from AMD donors when compared with RPE isolated from non-AMD donors (Fig. 7D).

Our *in vitro* data showed that DJ-1 is oxidized at C106 when the cells are exposed to oxidative stress. To check if oxDJ-1 is present in RPE cells *in vivo*, RPE lysates from AMD and non-AMD donors were probed for this modification with a specific antibody (Fig. 7B). oxDJ-1 was present in the RPE lysates from AMD donors at higher levels (Fig. 7B, lanes 6 to 10) when compared to the RPE lysates from non-AMD donors (Fig. 7B, lanes 1 to 5). DJ-1 immunoreactivity was normalized to the GAPDH content of the samples (Fig. 7C). Quantitation of these blots showed that oxDJ-1 immunoreactivity was ∼6 fold higher in RPE isolated from AMD donors when compared with RPE isolated from non-AMD donors (Fig. 7D).

We next immunohistologically examined the location of DJ-1 in RPE from non-AMD and AMD donor eyes with geographic atrophy (Fig. 7E to L) and isolated BM/choroid from the perimacula of AMD donors (Fig. 7M to P). Immunostaining of DJ-1 was detected mostly in the RPE nuclei (arrowheads) but also in the cytoplasm of non-AMD donors (Fig. 7F and H, arrows). Significantly more DJ-1 was detected through out the cytoplasm of RPE cells from two different AMD donors wit geographic atrophy (Fig. 7J and L, arrows). Interestingly, DJ-1 presence in RPE cells became less intense at distances away from the region of RPE atrophy (Figure S3). In addition, in BM/choroid isolated from two different AMD donors and drusen (insoluble aggregates localized underneath the RPE embodying the hallmark lesions of the disease, asterisks), in these samples were also specifically labeled with the DJ-1 antibody (Fig. 7N and P). In contrast, no significant DJ-1 labeling was observed when sections were reacted with the DJ-1 antibody, pre-absorbed with lysates from cells overexpressing exogenous DJ-1 (Fig. 7E, 7G, 7I, 7K, 7M and 7O). Our results showed that higher levels of DJ-1 and oxDJ-1 are present in RPE cells from AMD donors. In addition, we also reported that DJ-1 is also present in the hallmark lesion of this disease.

## Discussion

Previous reports showed that DJ-1 is ubiquitously expressed in numerous tissues including the pancreas, kidney, skeletal muscle, liver, placenta, heart and brain, with high expression in astrocytes of the frontal cortex and substantia nigra [9]. We recently reported the identification of DJ-1 peptides in both young and aged RPE lysates using proteomics together with confirmation of the localization of DJ-1 in RPE cells in rat retinas using immunohistochemistry [44]. Here, we described DJ-1 increased expression and redistribution to mitochondria of RPE cells under oxidative stress. In addition, we report that overexpression of full-length DJ-1, prior to exposure to oxidative stress, led to significant decrease in the generation of ROS. Most importantly, increased DJ-1 and oxDJ-1 were detected by Western blot in human RPE lysates from AMD donors. Finally, immunohistology detected DJ-1in isolated human BM/choroid and in drusen from AMD eyes.

Results reported here in RPE cultures under baseline and oxidative stress conditions are in agreement with previous reporting of the subcellular distribution of DJ-1. At the subcellular level, under baseline conditions, DJ-1 is found mostly in the cytoplasm [32] and to a lesser extent in the mitochondria [23] and nucleus [50]. Under conditions of oxidative stress, more DJ-1 redistributes to mitochondria and later to the nucleus, and this correlates with the ability of DJ-1 to confer neuroprotection [23].

Our results reporting increased levels of DJ-1 in RPE cells subjected to oxidative stress induced by incubation with H_2_O_2_ and 4-HNE suggest that DJ-1 functions as a sensor of cellular redox homeostasis, which reacts to oxidative stress by increasing DJ-1 content. Similar data was previously reported when experiments were carried out in other cell types under oxidative stress induced by several agents [30], [51], [52], [53], [54], [55], [56], [57].

DJ-1 has three C at amino acid numbers 53, 46 and 106, which can become oxidized upon oxidative stress and oxidation of C106 (oxDJ-1) is essential for DJ-1 to exert its full activities. In the present study we report the presence of oxDJ-1 in RPE cells in response to oxidative stress using antibodies that specifically recognize DJ-1 oxidized at C106 (oxDJ-1). Similar results have also been previously reported in other cells types and PD animal models [18], [47], [58], [59], [60]. DJ-1 has four Met (M) residues, which are also susceptible to oxidation in addition to the three C residues already cited. The oxidation of the M to the sulfoxide (1 O), and C to the sulfenic acid (1 O), sulfinic acid (2 Os) or sulfonic acid (3 Os) is expected to lead to increases in the number of O atoms in the protein ranging from four, if all four M residues are oxidized, and nine if all three C residues are fully oxidized, for a potential total of 13 additional oxygen atoms [18]. Interestingly, the molecular weight of the protein detected in RPE cells incubated with H_2_O_2_ displayed a higher molecular weight than the reported DJ-1 molecular weight (∼25 kDa). The meaning of this observation is not fully known but it may be related to the generation of several DJ-1 isoforms resulting from the oxidation of the M and C residues [18], [54], [61].

The importance of DJ-1 C oxidation is also highlighted in RPE cells overexpressing C to S DJ-1 mutant that are exposed to oxidative stress, which are unable to increase DJ-1 levels and redistribute DJ-1 intracellularly to mitochondria. Our results, together with previous reports, suggests that DJ-1 C oxidation is important for protein stabilization [61], [62], [63], [64] and mitochondria localization [23], [52], [65].

We demonstrated that overexpression of full-length but not the C to S mutant DJ-1 lead to significant decrease in the generation of intracellular ROS. The data suggests DJ-1 C oxidation –dependent elimination of ROS in cells under oxidative stress. Similar findings have been reported [52], [57], [66], [67], [68], [69].

We demonstrate here for the first time that DJ-1 levels are increased in RPE lysates from AMD donors and that DJ-1 immunolocalization in RPE is also increased in AMD donors displaying geographic atrophy. AMD is a multi-factorial disease with known established risk factors including age, cigarette smoking, family history, gender, high blood pressure, high fat diet and race [70], [71], [72]. However, our understanding of the detailed molecular mechanisms of the development of AMD still remains limited, and to date, there is no proficient cure or preventive treatment. Oxidative stress affecting the physiological function and leading to the death of the RPE cells is a major factor contributing to the pathogenesis of AMD [6], [7]. Therefore, limiting RPE oxidative stress may represent an effective way to slow or possibly reverse vision loss of patients due to diseases such as AMD. Indeed, several *in vitro* studies have shown that oxidative stress-related RPE cell death and dysfunction was improved when the cells were treated with antioxidants [73], [74], [75], [76]. Furthermore, a significant reduction toward retinal degeneration was reported in clinical trials involving AMD patients ingesting antioxidants such as lutein, zeaxanthin, zinc, vitamin C, and vitamin E [77], [78]. It is likely that the increased levels of DJ-1 in RPE lysates from AMD donors is related to increased oxidative stress in these RPE cells *in vivo*. Increased levels of DJ-1 were also reported in the brains of PD and Alzheimer’s disease (AD) patients [27].

The increased presence of oxDJ-1 was reported in corneal buttons from Fuchs endothelial corneal dystrophy patients [56]. In addition, the presence of several oxidized DJ-1 isoforms have been found in patients with PD [79] and in patients with AD [27]. These data suggest that the DJ-1 oxidation status modulates its functions and that deregulation of DJ-1 oxidation may lead to the onset of diseases such as AMD [48]. In the present study we detected the increased presence of oxDJ-1 in RPE lysates from AMD donors using an antibody that detects oxidation at C106. Future experiments will be required to fully understand the significance of our finding.

A high number of mitochondria are present in the RPE due to its high metabolic activity [80]. During oxidative phosphorylation, the mitochondria produce the majority of the cellular energy in the form of ATP and also generate ROS as well [81], [82]. A vicious circle of destruction takes place when ROS, including those produced in mitochondria by the electron transport chain, are generated leading to the preferred damage of mitochondrial genome (mtDNA). In turn, damaged mtDNA induces mitochondrial dysfunction with disturbance of oxidative phosphorylation and even higher production of ROS [2], [83]. Therefore, it is not surprising that mitochondrial dysfunctions leading to increased ROS generation and mtDNA damage and specific hapogroups have been implicated in the pathophysiology of AMD [84], [85], [86], [87], [88], [89], [90], [91], [92]. We have shown here that an increased association of DJ-1 with mitochondria is observed in cells under oxidative stress. Therefore, it is likely that DJ-1 is “over-oxidized” in RPE cells from AMD patients failing to associate with the mitochondria and protect RPE cells from oxidative stress. Further experiments should concentrate on the analysis of DJ-1 intracellular distribution and oxidative isoforms in AMD and non-AMD RPE.

We reported here that DJ-1 was detected in Bruch’s membrane and drusen isolated from two different AMD donors raising the question of how a cytoplasmic protein could be detected extracellularly. Previously, we [93], [94] and other groups [95], [96], [97], [98] reported that many of proteins found in drusen are normally found intracellularly in the RPE. These results suggest that RPE cells from AMD donors release intracellular proteins along their basal surface through a yet unknown mechanism where they become concentrated in drusen. DJ-1 is an intracellular protein. However, recently DJ-1 has emerged as a significant biomarker since its presence has been detected in the serum of gastric cancer [99], prostate carcinomas [100], pancreatic cancer [101], non-small cell lung cancer [102] and uveal melanoma [103] patients. Similarly, higher DJ-1 levels have been noted in nipple secretions from breast carcinoma patients [104]. Moreover, DJ-1 has been detected in cerebrospinal fluid from Parkinson’s Disease patients [105], [106], [107] and urine from hepatocellular carcinoma [108] patients. Future studies will be needed to investigate how RPE cells release DJ-1 and to determine if it can be detected in the serum of AMD patients.

In summary, due to the evidence presented here connecting DJ-1 to protection against oxidative stress, it is conceivable that manipulation of DJ-1 function may be used to protect RPE cells from the oxidative stress implicated in AMD pathology.

## Methods

### Ethics Statement

The immunocytochemistry and Western analysis of human isolate RPE and BM/choroid is exempt of IRB approval since the human tissue was obtained and used after deceased.

All animal work was conducted in compliance with the Animal Welfare Act and Public Health Services policies, and under the oversight and approval of the Cleveland Clinic Institutional Animal Care and Use Committee (IACUC, protocol number ARC 2010-0136). All efforts were made to minimize animal suffering.

### RPE Cell Cultures

Unless specified all media components were prepared in-house at the Cleveland Clinic cell culture lab core from commercially produced powders from Invitrogen, Sigma-Aldrich, and Caisson Labs.

The established cell line D407 [109], obtained as a gift from Dr. Richard Hunt (University of South Carolina School of Medicine, SC) was cultured at the temperature of 37°C as previously described [109]. B6-RPE07 mouse cells were cultured as previously described [45]. Briefly, cells were grown in Dulbecco's modified Eagle’s medium (DMEM) supplemented with 3% heat-inactivated fetal bovine serum, glutamax (Gibco), non-essential amino acids, and penicillin/streptomycin. To promote differentiation, B6-RPE and D407 cells, which have a very high rate of growth, were transferred to and cultured in serum-free epithelial medium for two passages (Quantum 286 for epithelia cells; PAA Laboratories Ltd.) before plating on laminin-(BD Biosciences, San Jose, CA) and collagen-coated Transwell inserts (Corning, Corning, NY), respectively. The human RPE cell line ARPE-19 was obtained from American Type Culture Collection (Manassas, VA, USA). The cells were maintained in DMEM/F12 1:1 containing 10% FBS and 5.5 mmol D-glucose in a humidified incubator at 37°C in 5% CO2. The medium was changed every 3–4 days. Polarized ARPE-19 cells were plated and cultured for 3 weeks on collagen-coated Transwell inserts in 1% FBS medium before using in experiments. Primary mouse retinal pigment epithelium was isolated as previously described [110]. Briefly, eyes from ∼2 week-old C57BL/6J mice were treated with 0.5 mg/ml bovine hyaluronidase (Sigma Chemical, St. Louis, MO) and 0.05 mg/ml of collagenase (Sigma-Aldrich) followed by and 0.1% trypsin (Difco-BD Biosciences, Sparks, MD) in buffer for 60 min each incubation to allow the mechanical separation of the neural retina and exposure of the RPE. Patches of RPE were peeled off manually from Bruch's membrane. To further dissociate the RPE patches, purified RPE cells were incubated with 0.05% trypsin/0.53 mM EDTA for 2 min at 37°C.

### Human Eye Tissue

Donor eyes were obtained from the Cleveland Eye Bank or through the Foundation Fighting Blindness Eye (FFB) Donor Program (Columbia, MD). Tissue from 22 different donors were analyzed including 12 samples from non-AMD donors and 17 from AMD donors many of which, had previously been described [111]. The donor ages ranged between 35 and 91 years and the interval between time of death and tissue processing varied between 4 and 35.5 hours. Eye bank records accompanying the donor eyes indicated whether the donor had AMD or no known eye diseases.

### Oxidative Stress Treatment

Monolayers were rinsed with warm PBS and incubated with PBS for 5 min at 37°C. Oxidative stress was induced by incubation with culture medium supplemented with 0–800 µM H_2_O_2_ for 1 and 24 hours. Alternatively, monolayers were stressed with 0–100 µM 4-HNE for 12 and 24 hours in SFM. Following this incubation, monolayers were washed with pre-warmed PBS and either fixed with 4% paraformaldehyde made in PBS and processed for immunofluorescence labeling or scraped and pelleted down to be processed for biochemistry analysis.

### Adenovirus Infection

ARPE-19 cells were cultured as previously described. The replication-defective adenovirus vectors Ad5CMVPARK7 (for expression of human DJ-1 under control of a human cytomegalovirus [CMV] promoter) and AdCMVPARK7. C to S (for expression of human DJ-1 with the cysteine at residues 46, 53 and 106 mutated to serine) were prepared and titered by Welgen Inc. (Worcester, MA) using PARK7 human cDNA clone obtained from Origene (SC115623, Rockville, NY). To transduce cells, adenoviruses were mixed with transduction medium (20 mM Hepes-buffered DMEM containing 0.2% BSA) and incubated with cells for 2 h at a concentration of 5×10^6^ plaque forming units (pfu)/cell unless specified in text. Cells on Transwells had viruses added to both apical and basal surface. After infection, transduction medium was replaced by normal culture medium and cells were returned to incubator. Two days after adenovirus transduction, cells were washed with pre-warmed PBS and either fixed with 4% paraformaldehyde made in PBS and processed for immunofluorescence or scraped and pelleted down to be processed for biochemistry analysis. Alternatively, cells were exposed to oxidative stress as described above and then processed for immunofluorescence or biochemistry analysis.

### Cell Loading with Fluorescent Probe to Detect Reactive Oxygen Species (ROS) Production

ARPE-19 monolayers were plated on glass coverslips and cultured as described above. Monolayers infected or not with adenoviruses as described above, were subjected to oxidative stress as described above. A 10 µM stock solution of CM-H_2_DCFDA (Molecular Probes, C6827) was prepared in DMSO immediately before use. Monolayers were loaded by a 15-min incubation with 10 µM DCFH-DA at room temperature in the dark. Following incubation, monolayers were washed with pre-warmed PBS at room temperature and carefully mounted immediately on the microscope slide in vectashield. Labeled cells were analyzed using a Leica laser scanning confocal microscope (TCS-SP2, Leica, Exton, PA). Cellular images were obtained by difference interference contrast (DIC).

### Immunofluorescence of Cells

RPE monolayers on Transwells were fixed in 4% paraformaldehyde for 30 minutes at 4°C. Cells were blocked in PBS +1% BSA and incubated overnight at 4°C with poyclonal antibody directed against DJ-1 (NB300-270, 1∶750; Novus), and DJ-1 oxidized at C106 (oxDJ-1, HCA024, 1∶50; AbD serotec, Oxford, UK) and monoclonal antibody directed against OxPhos Complex IV subunit I (COX IV, 459600, 1∶500; Invitrogen). Alexa Fluor488, Alexa Fluor594 and anti-Myc Tag Alexa Fluor488 secondary antibodies were added at room temperature for 1 hour (1∶1000; Molecular Probes and 1∶500; Millipore) and cell nuclei were labeled with TO-PRO®-3 iodide (1∶5000; Molecular Probes). Monolayers were analyzed using a Leica laser scanning confocal microscope. A series of 1 µm *xy* (*en face*) sections were collected. Each individual *xy* image of the retinas stained represents a three-dimensional projection of the entire cryosection (sum of all images in the stack). Microscopic panels were composed using Adobe Photoshop CS3 (Adobe, San Jose, CA).

### Immunohistology of Human Donor Tissue

To determine the localization of DJ-1 in RPE cells from non–AMD and AMD eyes and BM/choroid isolated from AMD donors, immunohistochemical assays were performed using cryosections in the peri-macular area. Isolated BM/choroid strips were isolated from the eyecups as previously described [94]. Eye pieces of retina-RPE-choroid were cut and fixed by immersion in 4% paraformaldehyde made in PBS overnight at 4°C, quenched with 50 mM NH4Cl made in PBS for 1 h at 4°C, infused successively with 10% and 20% sucrose made in the same buffer and with Tissue-Tek “4583” (Miles Inc., Elkhart, IN) as previously described [111]. Cryosections (8 µm) were cut on a cryostat HM 505E (Microm, Walldorf, Germany) equipped with a CryoJane Tape-Transfer system (Instrumedics, Inc., Hackensack, NJ, USA). Cryosections were washed, and processed for antigen retrieval in pre-heated Trilogy (Cell Marque, Rocklin, CA**)** by incubation for 30 min. in a steamer followed by transfer to room temperature for 20 min. to allow cryosections to cool down. Sections were blocked with PBS +1% BSA and probed with the DJ-1 antibody (TA301239, 1∶750, Origene) overnight at 4°C followed by labeling with Vectastain Elite ABC reagent (Vector Laboratories, Inc., Burlingame, CA) according to the manufacture’s directions. Negative controls were pre-absorbed overnight in the rotator with 5 µg of lysates of HEK293 cells overexpressing PARK7. Labeling was detected through incubation with ImmPACT VIP peroxidase substrate (Vector Laboratories) according to the manufacturer’s instructions. Slides were mounted in cytoseal (Richard-Allan-Scientific, Kalamazoo, MI). The sections were examined with a Zeiss AxioImager.Z1 light microscope and the images were digitized using a Zeiss AxioCam MRc5 camera.

### Western Blot Analysis

RPE cells were solubilized in RIPA buffer (0.1% SDS, 1% Triton X100, 1% deoxycholate, 0.15 M NaCl, 2 mM EDTA, 25 mM Tris pH 7.4) supplemented with a cocktail of protease and phosphatase inhibitors (Sigma Chemical Co., St. Louis, MO). Total RPE lysates (20 µg protein) were resolved by SDS-PAGE on 4–20% Novex®-Tris-Glycine gel (Invitrogen Corporation, Carlsbad, CA) and electro-transferred to Immobilon PVDF membranes (Millipore, Bedford, MA). Membranes were blocked with HyBLOCKER liquid blocking reagent (Denville Scientific Inc., Metuchen, NJ) for 30 min. and incubated overnight in the same solution with antibodies to DJ-1 (NB300-270, 1∶2000; Novus), DJ-1 oxidized at C106 (oxDJ-1, HCA024, 1∶200; AbD serotec), GAPDH (ab9484, 1∶1000; Abcam). Protein detection was performed with secondary antibodies conjugated to peroxidase and visualized using ECL Plus Western Blotting detection reagent (GE Healthcare Bio-Sciences Corp, Piscataway, NJ). PVDF membranes were exposed to film. Films were scanned and figures were composed using Adobe Photoshop CS3. Signal intensities were quantified using ImageJ 1.43u (http://rsb.info.nih.gog/ij).

## Supporting Information

Figure S1 **Oxidative stress induced by 4-HNE increases DJ-1 levels in RPE cells.** ARPE-19 and D407 monolayers were treated with increasing concentrations (0 to 100 µM) of 4-HNE for 12 hrs (A) harvested, and analyzed by immunoblot assay with DJ-1 antibody (upper panel). Each lane contained 20 µg of protein. Protein loadings were confirmed in replicate blots probed with GAPDH (lower panel). A dose response of ARPE-19 (A, lanes 1 to 6) and D407 (A, lanes 7 to 12) is observed when cells are exposed to increasing concentrations of 4-HNE for 12 hrs. Quantitation of these blots showed that DJ-1 immunoreactivity was 1.5 and 1.4 fold higher in ARPE-19 incubated with 5 and 10 µM HNE and up to 2.1 fold in D407 cells incubated with 25 µM HNE when compared with control cell RPE cultures (B). Plotted data represent the intensity values for each band normalized to GAPDH signal and compared to the intensity of the control, untreated cells (lanes 1, 7). Red columns = ARPE-19; blue columns = D407 cells. Data is expressed as mean relative signal intensity ± SEM (n = 3). Asterisks denote statistical significance compared with control untreated cells (*p = 0.0097 and **p<0.0001 in the ARPE-19 and *p = 0.0006, **p = 0.0499, ***p = 0.0020, ****p<0.0001 in D407 cells).
(TIF)

Figure S2 **Oxidative stress-dependent translocation of DJ-1 into mitochondria**. Representative confocal micrographs of B6-RPE07 monolayers plated on glass coverslips and labeled with antibodies to DJ-1 (A, D) and the mitochondrial staining MitoTracker (B, E). Cell nuclei were labeled with TO-PRO-3. Under baseline conditions, there is very little colocalization between DJ-1 and MitoTracker, as observed in overlaid images (C). Upon oxidative stress induced by incubation with 200 µM H_2_O_2_ for 18 hrs, the diffused cytoplasmic DJ-1 staining disappears. Moreover, in overlaid images a pronounced mitochondrial staining for DJ-1 is apparent when cells are exposed to oxidative stress (F, arrows). Scale bar = 20 µm.
(TIF)

Figure S3 **Increased levels of DJ-1 in region of RPE atrophy in AMD donor.** Cryosections of non-AMD (A) and AMD (B, C) donors with geographic atrophy were labeled with DJ-1 antibody. DJ-1 labeling was detected mostly in the RPE nuclei (arrowheads) but also in the cytoplasm (A, arrows) of non-AMD donors; labeling was also observed in the choriocapillaris (A, double arrowheads). Significantly more DJ-1 was detected all over the cytoplasm of RPE cells (B, arrows) and choriocapillaris (B, double arrowheads) from an AMD donor with geographic atrophy in the atrophic region; labeling was also significantly more intense in the choriocapillaris in this region. DJ-1 labeling of a druse (B, asterisks) is also observed. Interestingly, in this same AMD donor eye, at distances away from the region of RPE atrophy, DJ-1 immunoreactivity was similar to that observed in the RPE normal control eyes (C). Scale bars = 10 µm.
(TIF)
