# Correction: DJ-1-Dependent Regulation of Oxidative Stress in the Retinal Pigment Epithelium (RPE)

**DOI:** 10.1371/journal.pone.0104292

**Published:** 2014-07-28

**Authors:** The PLOS ONE Staff

## Notice of Republication

This article was republished on July 10, 2014, due to Figure 2 being erroneously removed from the PDF. The publisher apologizes for this error.

## Supporting Information

File S1 Originally published, uncorrected article.
(pdf)

File S2 Republished, corrected article.
(pdf)
