# Correction: DJ-1-Dependent Regulation of Oxidative Stress in the Retinal Pigment Epithelium (RPE)

**DOI:** 10.1371/journal.pone.0185834

**Published:** 2017-10-02

**Authors:** Karen G. Shadrach, Mary E. Rayborn, Joe G. Hollyfield, Vera L. Bonilha

The authors would like to correct Figs 2 and 4, as errors were introduced in the preparation of these figures for publication. The GAPDH control load in Fig 2A (D407 +H_2_O_2_ 1h) had been duplicated by mistake in Fig 2B (ARPE-19 + H_2_O_2_ 18hr). These same ARPE-19 +H_2_O_2_ blots were stripped and re-probed in Fig 4 with the oxDJ-1 antibody. The authors checked the original images obtained and the GAPDH panels in Figs 2 and 4 were corrected. The correct versions of Figs 2 and 4 are provided here, along with their corrected figure legends. The authors confirm that these changes do not alter their findings. The authors have provided the underlying images as Supporting Information.

**Fig 2:**
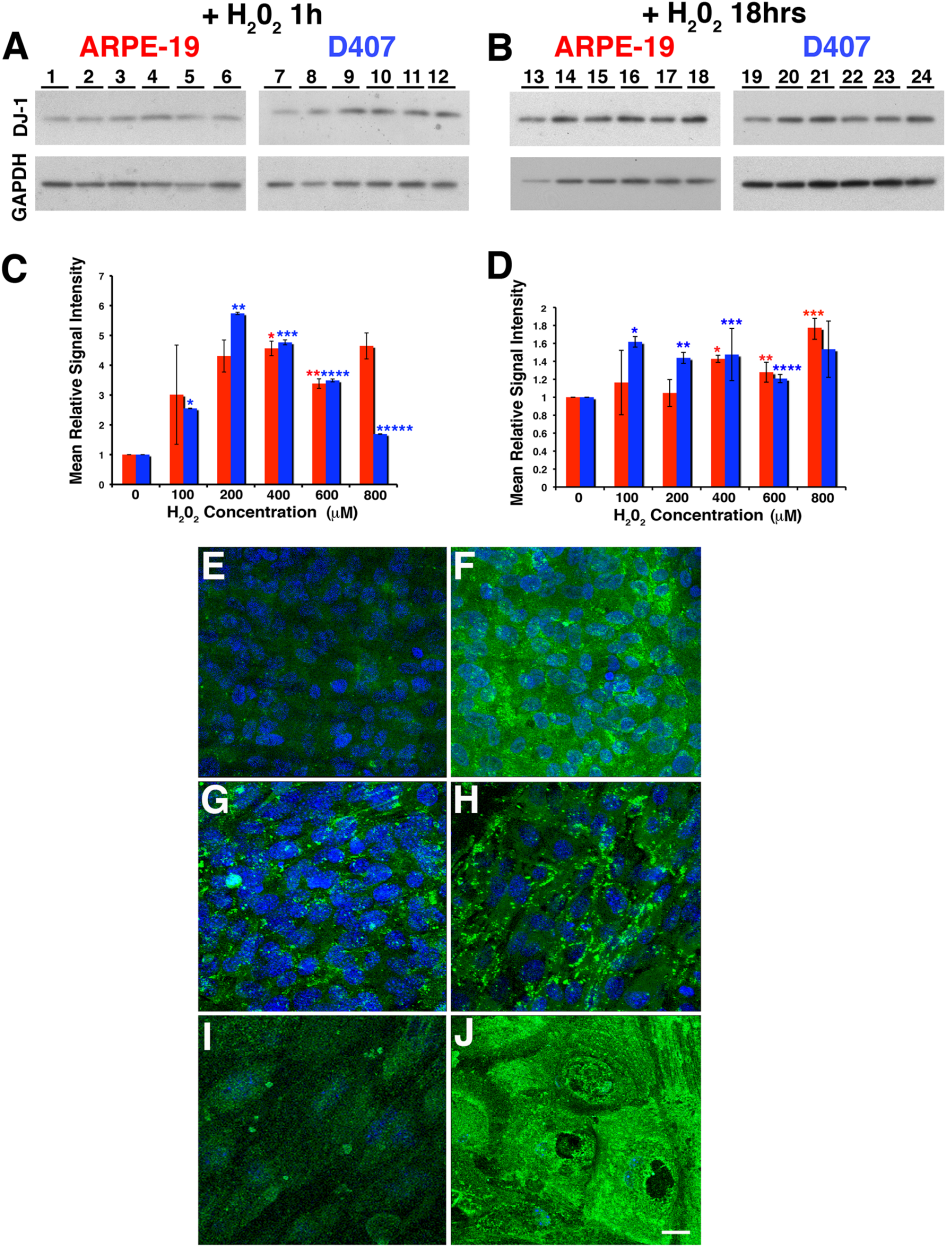
Oxidative stress induced by H_2_O_2_ increases DJ-1 levels and leads to intracellular redistribution of DJ-1 in RPE cells. ARPE-19 and D407 monolayers were treated with increasing concentrations (0 to 800μM) of H_2_O_2_ for 1hr (A) and 18hrs (B), harvested, and analyzed by immunoblot assay with DJ-1 antibody (upper panel). Each lane contained 20 μg of protein. Protein loadings were confirmed in replicate blots probed with GAPDH (lower panel). A representative Western is shown. A dose response of ARPE-19 (A, lanes 1 to 6) and D407 (A, lanes 7 to 12) is observed when cells are exposed to increasing concentrations of H_2_O_2_ for 1hr. Quantitation of these blots showed that DJ-1 immunoreactivity was 5.0 and 3.6 fold higher in ARPE-19 incubated with 400 and 600 μM H_2_O_2_ and up to 5.7 fold higher in D407 cells incubated with 200 μM H_2_O_2_ when compared with control cell RPE cultures (C). Plotted signals represent the intensity for each band normalized to GAPDH signal and compared to the intensity of the control, untreated cells (lanes 1, 7, 13, 19). Red columns = ARPE-19; blue columns = D407 cells. Data is expressed as mean relative signal intensity ± SEM (n = 3). Asterisks denote statistical significance compared with control untreated cells (*p = 0.0160 and **p = 0.0145 in the ARPE-19 and *p< 0.0001, **p< 0.0001, ***p = 0.0005, ****p = 0.0004 and p***** = 0.0001 in D407 cells). Similarly, both ARPE-19 (Fig. B, lanes 13 to 18) and D407 (Fig. 2b, lanes 19 to 24) also displayed a dose response when cells were exposed to increasing concentrations of H_2_O_2_ for 18hrs. Quantitation of these blots showed that DJ-1 immunoreactivity was 1.4, 1,3 and 1.8 fold higher in ARPE-19 incubated with 400 to 800 mM H_2_O_2_ and up to 1.6 fold higher in D407 cells incubated with 100 to 800 μM H_2_O_2_ when compared with control cell RPE cultures (D). Asterisks denote statistical significance compared with control untreated cells (*p = 0.0010, **p = 0.0146 and ***p = 0.0185 in the ARPE-19 and *p = 0.0005, **p = 0.0020, ***p = 0.0177 and ****p = 0.0103 in D407 cells). **E-J.** Confocal immunofluorescence staining of ARPE-19 (E, F), B6-RPE07 (G, H) and mouse primary RPE cultures (I, J) fixed before extraction with Triton X-100 and labeling with DJ-1 antibody. Cell nuclei were labeled with TO-PRO-3. Observations demonstrated that at baseline conditions, DJ-1 is diffused in the cytoplasm (arrows) and nuclei (*) of polarized RPE cells (E, G, I). With 18 hrs of exposure to 400 μM H_2_O_2_ (F, H, J), the diffused cytoplasmic DJ-1 staining disappears and pronounced aggregated perinucler staining (arrowheads) for DJ-1 is apparent. Scale bar = 10μm.

**Fig 4:**
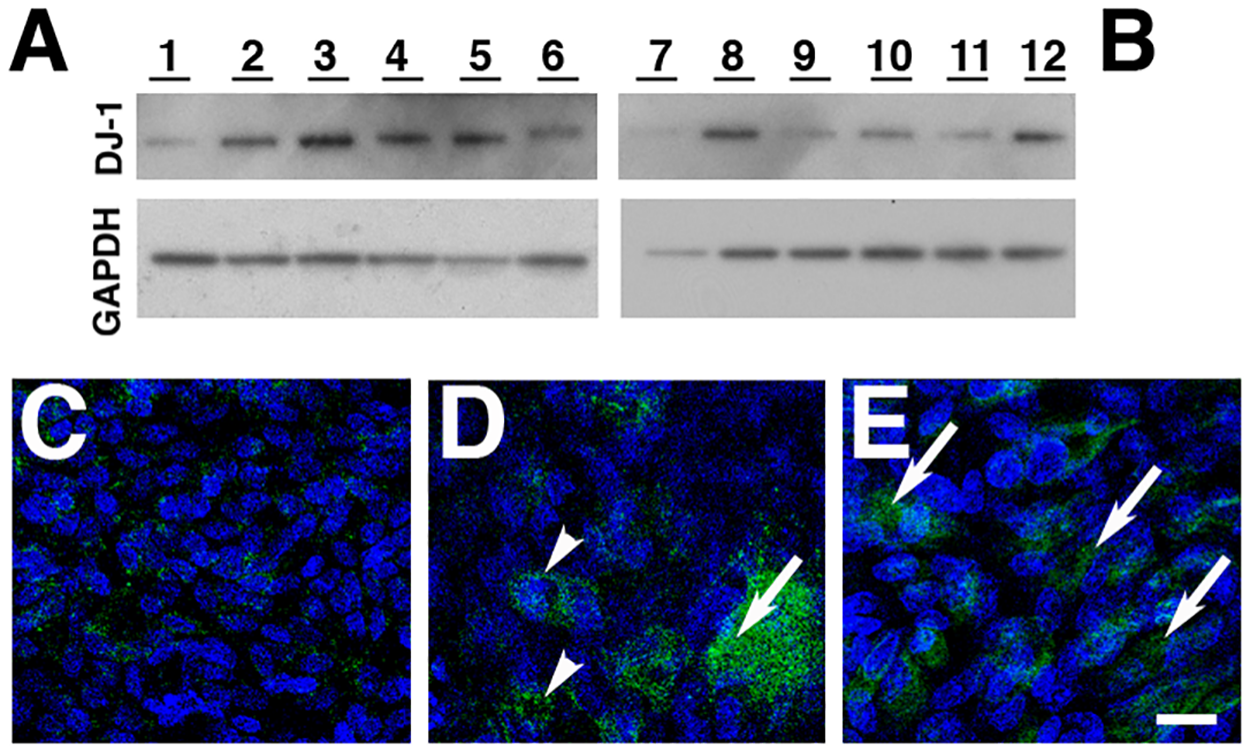
Presence of oxDJ-1 in RPE cells subjected to oxidative stress. ARPE-19 monolayers were treated with increasing concentrations (0 to 800 μM) of H_2_O_2_ for 1hr (A) and 18hs (B), harvested, and analyzed by immunoblot assay with oxDJ-1 antibody (upper panel). Protein loadings were confirmed in replicate blots probed with GAPDH (lower panel); the same blots displayed in Fig 2A and 2B were stripped and re-probed with oxDJ-1 antibody. Each lane contained 20 μg of protein. A dose response is observed when cells are exposed to increasing concentrations of H_2_O_2_ for 1h (A, lanes 1 to 6) and 18hrs (B, lanes 7 to 12). Confocal immunofluorescence staining of baseline ARPE-19 cultures (C) fixed before extraction with Triton X-100 and labeling with oxDJ-1 antibodies revealed absence of oxDJ-1. However, oxDJ-1 is observed in the cytoplasm (arrows) and perinuclear area (arrowheads) of RPE cells exposure to 400 μM H_2_O_2_ for 1h (D) and 18hrs (E). Cell nuclei were labeled with TO-PRO-3. Scale bar = 20μm.

## Supporting information

S1 Dataset Uncropped blots for Figs 2 and 4. (ZIP)
