# Supplementary figures and images for: DJ-1-Dependent Regulation of Oxidative Stress in the Retinal Pigment Epithelium (RPE)
Source: PLoS One. 2013 Jul 2;8(7):e67983. doi: 10.1371/journal.pone.0067983 (PMC3699467; doi:10.1371/journal.pone.0067983)

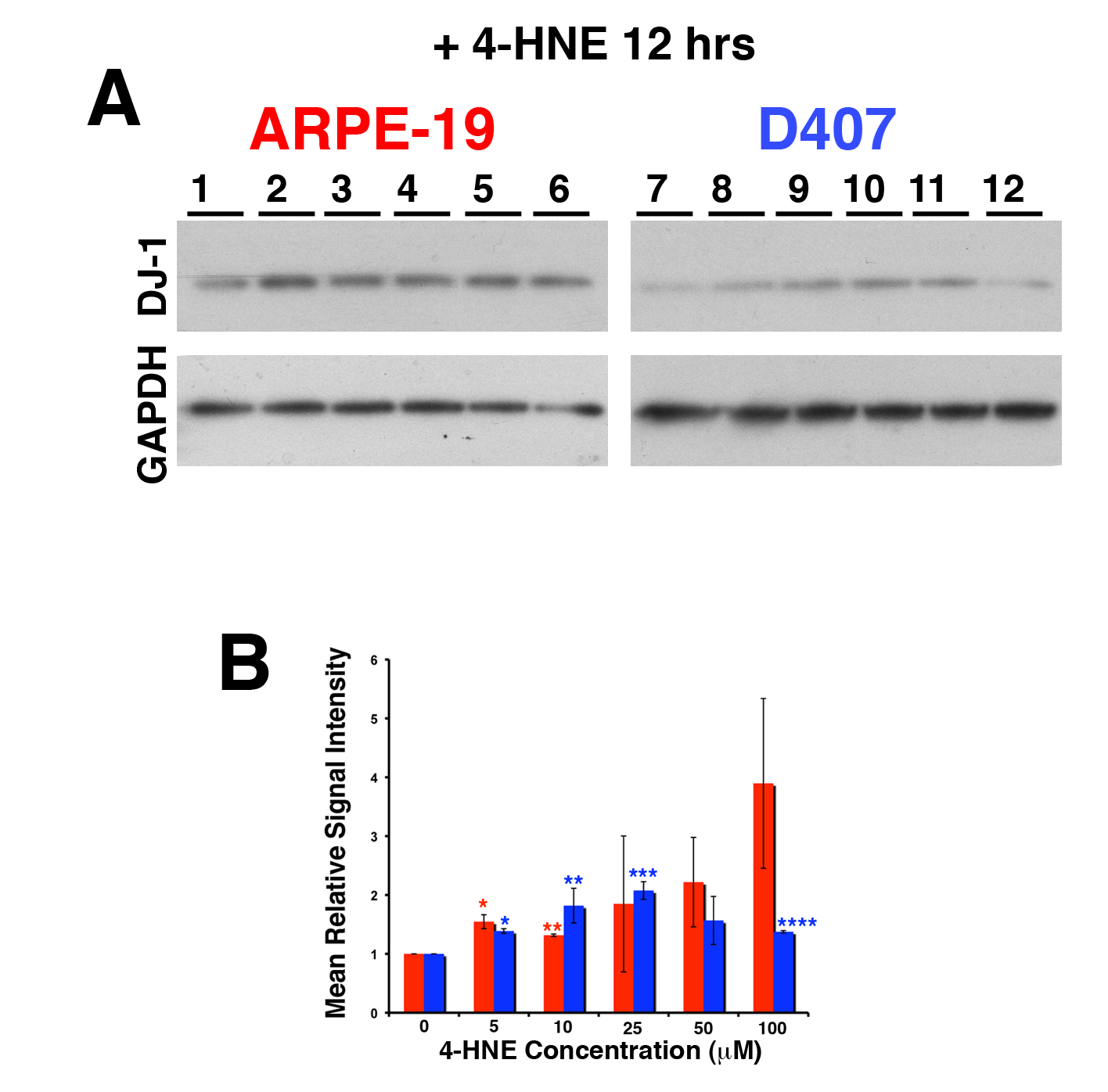

Supplement: Figure S1 — Oxidative stress induced by 4-HNE increases DJ-1 levels in RPE cells. ARPE-19 and D407 monolayers were treated with increasing concentrations (0 to 100 µM) of 4-HNE for 12 hrs (A) harvested, and analyzed by immunoblot assay with DJ-1 antibody (upper panel). Each lane contained 20 µg of protein. Protein loadings were confirmed in replicate blots probed with GAPDH (lower panel). A dose response of ARPE-19 (A, lanes 1 to 6) and D407 (A, lanes 7 to 12) is observed when cells are exposed to increasing concentrations of 4-HNE for 12 hrs. Quantitation of these blots showed that DJ-1 immunoreactivity was 1.5 and 1.4 fold higher in ARPE-19 incubated with 5 and 10 µM HNE and up to 2.1 fold in D407 cells incubated with 25 µM HNE when compared with control cell RPE cultures (B). Plotted data represent the intensity values for each band normalized to GAPDH signal and compared to the intensity of the control, untreated cells (lanes 1, 7). Red columns = ARPE-19; blue columns = D407 cells. Data is expressed as mean relative signal intensity ± SEM (n = 3). Asterisks denote statistical significance compared with control untreated cells (*p = 0.0097 and **p<0.0001 in the ARPE-19 and *p = 0.0006, **p = 0.0499, ***p = 0.0020, ****p<0.0001 in D407 cells). (TIF) [file pone.0067983.s001.tif]

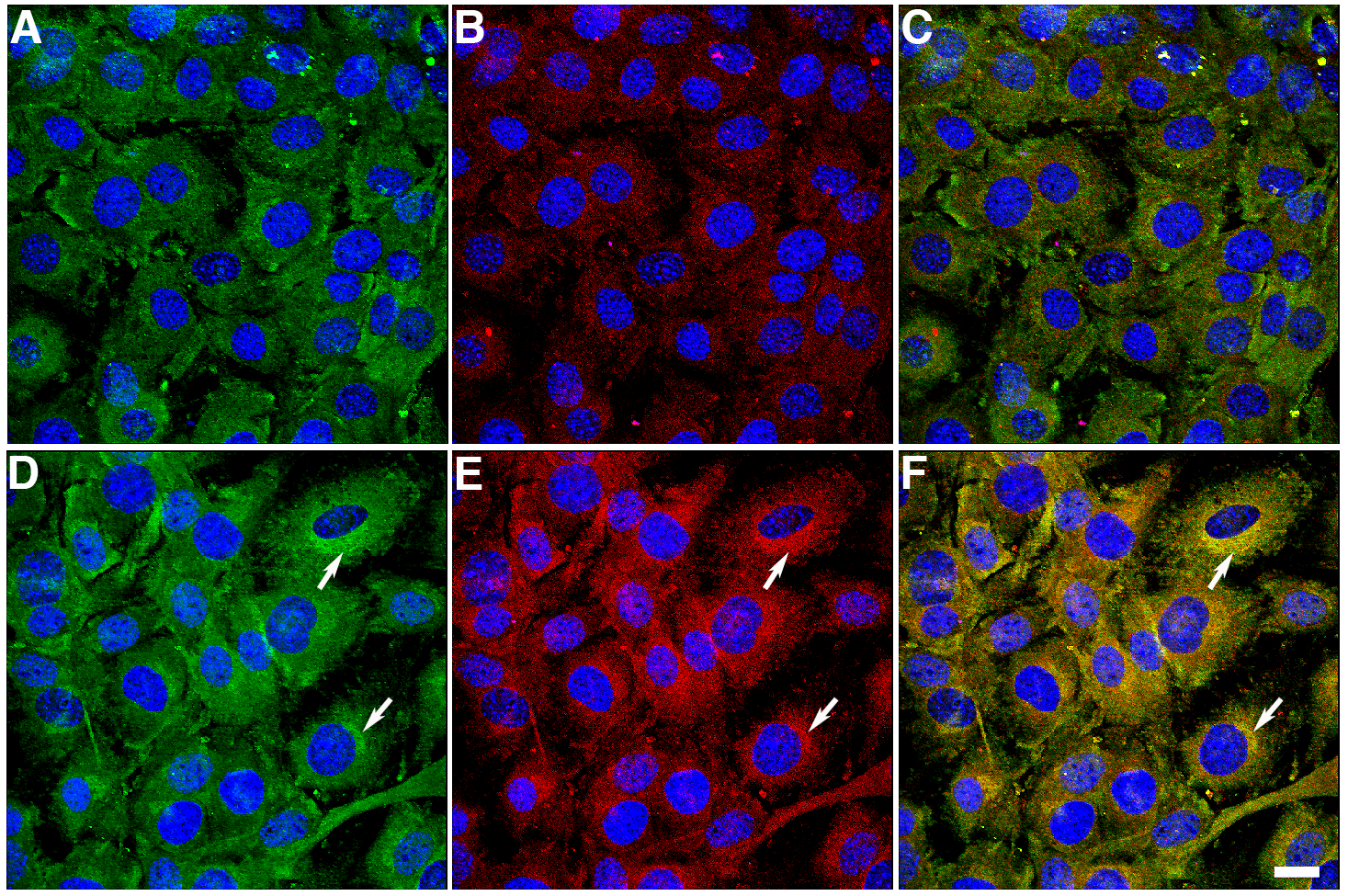

Supplement: Figure S2 — Oxidative stress-dependent translocation of DJ-1 into mitochondria. Representative confocal micrographs of B6-RPE07 monolayers plated on glass coverslips and labeled with antibodies to DJ-1 (A, D) and the mitochondrial staining MitoTracker (B, E). Cell nuclei were labeled with TO-PRO-3. Under baseline conditions, there is very little colocalization between DJ-1 and MitoTracker, as observed in overlaid images (C). Upon oxidative stress induced by incubation with 200 µM H2O2 for 18 hrs, the diffused cytoplasmic DJ-1 staining disappears. Moreover, in overlaid images a pronounced mitochondrial staining for DJ-1 is apparent when cells are exposed to oxidative stress (F, arrows). Scale bar = 20 µm. (TIF) [file pone.0067983.s002.tif]

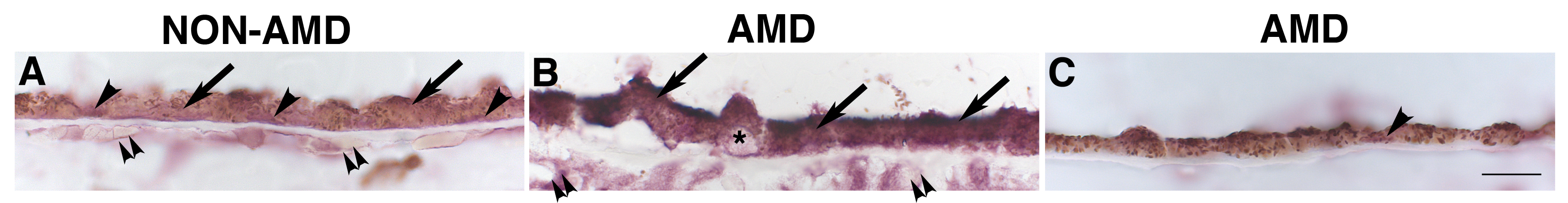

Supplement: Figure S3 — Increased levels of DJ-1 in region of RPE atrophy in AMD donor. Cryosections of non-AMD (A) and AMD (B, C) donors with geographic atrophy were labeled with DJ-1 antibody. DJ-1 labeling was detected mostly in the RPE nuclei (arrowheads) but also in the cytoplasm (A, arrows) of non-AMD donors; labeling was also observed in the choriocapillaris (A, double arrowheads). Significantly more DJ-1 was detected all over the cytoplasm of RPE cells (B, arrows) and choriocapillaris (B, double arrowheads) from an AMD donor with geographic atrophy in the atrophic region; labeling was also significantly more intense in the choriocapillaris in this region. DJ-1 labeling of a druse (B, asterisks) is also observed. Interestingly, in this same AMD donor eye, at distances away from the region of RPE atrophy, DJ-1 immunoreactivity was similar to that observed in the RPE normal control eyes (C). Scale bars = 10 µm. (TIF) [file pone.0067983.s003.tif]
