# Supplementary material for: Correction: DJ-1-Dependent Regulation of Oxidative Stress in the Retinal Pigment Epithelium (RPE)
Source: PLoS One. 2014 Jul 28;9(7):e104292. doi: 10.1371/journal.pone.0104292 (PMC4113426; doi:10.1371/journal.pone.0104292)
Supplement: File S1 — Originally published, uncorrected article. (pdf) [file pone.0104292.s001.pdf]

DJ-1-Dependent Regulation of Oxidative Stress in the Retinal Pigment Epithelium (RPE)

Karen G. Shadrach, Mary E. Rayborn, Joe G. Hollyfield, Vera L. Bonilha*

Department of Ophthalmic Research, The Cole Eye Institute, Cleveland Clinic Lerner College of Medicine, Cleveland, Ohio, United States of America

Abstract

Background: DJ-1 is found in many tissues, including the brain, where it has been extensively studied due to its association with Parkinson's disease. DJ-1 functions as a redox-sensitive molecular chaperone and transcription regulator that robustly protects cells from oxidative stress.

Methodology: Retinal pigment epithelial (RPE) cultures were treated with H_2O_2 for various times followed by biochemical and immunohistological analysis. Cells were transfected with adenoviruses carrying the full-length human DJ-1 cDNA and a mutant construct, which has the cysteine residues at amino acid 46, 53 and 106 mutated to serine (C to S) prior to stress experiments. DJ-1 localization, levels of expression and reactive oxygen species (ROS) generation were also analyzed in cells expressing exogenous DJ-1 under baseline and oxidative stress conditions. The presence of DJ-1 and oxidized DJ-1 was evaluated in human RPE total lysates. The distribution of DJ-1 was assessed in AMD and non-AMD cryosectionss and in isolated human Bruch's membrane (BM)/choroid from AMD eyes.

Principal Findings: DJ-1 in RPE cells under baseline conditions, displays a diffuse cytoplasmic and nuclear staining. After oxidative challenge, more DJ-1 was associated with mitochondria. Increasing concentrations of H_2O_2 resulted in a dose-dependent increase in DJ-1. Overexpression of DJ-1 but not the C to S mutant prior to exposure to oxidative stress led to significant decrease in the generation of ROS. DJ-1 and oxDJ-1 intensity of immunoreactivity was significantly higher in the RPE lysates from AMD eyes. More DJ-1 was localized to RPE cells from AMD donors with geographic atrophy and DJ-1 was also present in isolated human BM/choroid from AMD eyes.

Conclusions/Significance: DJ-1 regulates RPE responses to oxidative stress. Most importantly, increased DJ-1 expression prior to oxidative stress leads to decreased generation of ROS, which will be relevant for future studies of AMD since oxidative stress is a known factor affecting this disease.

Citation: Shadrach KG, Rayborn ME, Hollyfield JG, Bonilha VL (2013) DJ-1-Dependent Regulation of Oxidative Stress in the Retinal Pigment Epithelium (RPE). PLoS ONE 8(7): e67983. doi:10.1371/journal.pone.0067983

Editor: Xianglin Shi, University of Kentucky, United States of America

Received March 18, 2013; **Accepted** May 23, 2013; **Published** July 2, 2013

Copyright: © 2013 Shadrach et al. This is an open-access article distributed under the terms of the Creative Commons Attribution License, which permits unrestricted use, distribution, and reproduction in any medium, provided the original author and source are credited.

Funding: Supported by NIH grants EY014240, Research Center Grants from The Foundation Fighting Blindness and Research to Prevent Blindness. The funders had no role in study design, data collection and analysis, decision to publish, or preparation of the manuscript.

Competing Interests: The authors have declared that no competing interests exist.

* E-mail: bonilhav@ccf.org

Introduction

The retinal pigment epithelium (RPE) constitutes a monolayer of cuboidal cells. Its apical surface faces a very complex extracellular matrix called the interphotoreceptor matrix (IPM) that also surrounds the photoreceptor cells projecting from the outer retina. The RPE basal surface faces the underlying Bruch's membrane (BM) [1]. The RPE exhibit a number of highly specialized functions, including phagocytosis of shed tips of photoreceptor outer segments, directional transport of nutrients into and removal of waste products from photoreceptor cells, optimization of ion concentrations in the surrounding tissues, elimination of fluid from the IPM, and visual pigment regeneration and transport which, is essential to retinal homeostasis and vision.

The RPE cells live under chronic oxidative stress because of the lifelong exposure to light, high oxygen consumption and high oxygen partial pressure from the underlying choriocapillaris. In addition, phagocytosed shed photoreceptor outer segments are enriched in polyunsaturated fatty acids such as docosahexaenoate (DHA) and retinoids, which are highly susceptible to lipid peroxidation and fracgmentation leading to lipid peroxide–derived protein modifications [2,3]. Finally, phagocytosis itself increases oxidative stress resulting in the generation of endogenous reactive oxygen species (ROS) [4,5]. This hostile oxidative environment is thought to contribute to retinal disease. Indeed, it was suggested that oxidative stress affecting the physiological function and leading to focal loss of the RPE cells is a major factor contributing to geographic atrophy, and vision loss in the elderly blinding disease age-related macular degeneration (AMD) [6,7]. However, it remains to be determined why the initial retinal degeneration occurs and how degeneration processes progress as a result of continued oxidative insults.

Deletion or homozygous mutations of DJ-1 gene (*PARK7* locus) have been shown to cause early-onset autosomal recessive Parkinson's disease (PD) [8]. The DJ-1 gene encodes a highly conserved protein with 189 amino acids and a molecular weight of ~20 kDa. DJ-1 has been implicated in diverse cellular processes,

including cellular transformation and tumorigenesis [9,10,11], transcriptional regulation [12,13,14,15], androgen receptor signaling [16], chaperone [17,18], spermatogenesis [19], and oxidative stress response [20,21,22].

Oxidative stress occurs when there is an imbalance between the biological processes that produce ROS and the protective mechanisms within cells that scavenge ROS. Several studies have demonstrated that DJ-1 robustly protects cells from oxidative stress through distinct cellular pathways [20,21,23,24,25,26,27,28,29]. DJ-1 can eliminate H_2O_2 by becoming oxidized itself and thus functioning as a scavenger of ROS [20,21,30,31]. It was also proposed that some of the protective actions of DJ-1 might occur at the transcriptional level [32]. DJ-1 binds to PIAS proteins, a family of SUMO-1 ligases that modulate the activity of various transcription factors [16]. Wild-type DJ-1 sequesters the death protein Daxx in the nucleus, preventing it from binding and activating its effector kinase, apoptosis signal-regulating kinase 1 (Ask1) in the cytoplasm [33]. Others showed that DJ-1 is a transcriptional co-activator that interacts with the nuclear proteins p54nrb and PSF [34] again to protect against apoptosis. DJ-1 also stabilizes the antioxidant transcriptional master regulator nuclear factor erythroid-2 related factor 2 (Nrf2) [15,35] by preventing association with its inhibitor protein Keap1. Therefore, DJ-1 may act as a transcriptional co-factor that regulates the response to oxidative stress. DJ-1 has also been reported to confer protection against endoplasmic reticulum (ER) stress, proteasomal inhibition, and toxicity induced by overexpression of Pael-R [36]. Recently reported data showed that the apparently pleiotropic roles of DJ-1 seem to be related to the single function of binding multiple mRNA transcripts with a GG/CC-rich sequence [37]. More recently it was shown that DJ-1 plays a role in maintenance of mitochondria structure by counteracting the mitochondrial impairment induced by the tumour suppressor protein p53 [38]. Moreover, overexpression of the gene encoding DJ-1 protects against oxidative injury whereas knocking down the expression by RNAi enhances susceptibility to oxidative stress [21,36,39,40,41,42,43]. Thus, DJ-1 may play a crucial role both sensing and conferring protection against a range of oxidative stressors, through multiple mechanisms.

Previously, we have identified DJ-1 peptides in both young and aged RPE lysates using a proteomic approach [44]. In this study, we describe DJ-1 expression, distribution and function in RPE cells under baseline conditions and following oxidative stress. We also analyze DJ-1 and oxDJ-1 levels in human RPE lysates from non-AMD and AMD donors. Finally, we describe DJ-1 distribution in the RPE from non-AMD and AMD donors with geographic atrophy, and in isolated human BM/choroid (with drusen) from AMD eyes. Adaptation to changes in oxidative environments is critical for the survival of retina and the RPE. Therefore, knowledge of the DJ-1 function in oxidative stress in the RPE will provide insight into biochemical processes that support and maintain vision in physiological and pathological conditions.

Results

DJ-1 Expression and Distribution in RPE Cell Cultures

Recently, we identified DJ-1 peptides in both young and aged RPE lysates using proteomic analysis [44]. To decipher the molecular mechanism of DJ-1 function we decided to use several previously characterized RPE cell cultures. One was the newly characterized B6-RPE07 mouse RPE cell line, capable of displaying a polarized phenotype when cultured in a serum free epithelial medium and plated on collagen-coated Transwells [45]. We also used two human RPE cell lines: ARPE-19 and D407. We also used mouse primary RPE cultures, which morphologically resemble RPE cells *in vivo* due to their elaboration of apical microvilli and basal infoldings on their surfaces [46]. The initial characterization of these four cell lines analyzed the expression of DJ-1 by Western analysis (Fig. 1A and B) and immunofluorescence (Fig. 1C to F). A major band of ~25 kDa was observed in the extracts of all the RPE cell lines (Fig. 1A, lanes 1 to 3) and mouse primary RPE (Fig. 1A, lane 5) when compared to extracts from mouse brain (Fig. 1A, lane 4). Immunoblots of RPE lysates obtained from all cell cultures demonstrated heterogeneity in the levels of expression of DJ-1 when compared to the expression of the loading control protein GAPDH (Fig. 1B). DJ-1 expression in mouse primary RPE lysates displayed the highest levels of expression of DJ-1 among the RPE cells tested (Fig. 1A, lane 5). Confocal microscopy *en face* examination of paraformaldehyde-fixed monolayers grown on polycarbonate filters revealed that under baseline conditions, DJ-1 displays a diffuse cytoplasmic and, in some cells, nuclear staining (Fig. 1C to F, arrows) in all the RPE cell lines analyzed. DJ-1 analysis in polarized RPE monolayers established that although each one of the RPE cell cultures have different levels of DJ-1, they all display similar subcellular distribution of DJ-1.

DJ-1 Expression in RPE Cells Subjected to Oxidative Stress

To evaluate the role of oxidative stress on DJ-1 expression, RPE cultures were plated, incubated with H_2O_2 and the expression and distribution of DJ-1 was analyzed (Fig. 2). A representative Western is shown. A dose response relating DJ-1 expression in ARPE-19 (Fig. 2A, lanes 1 to 6) and D407 (Fig. 2A, lanes 7 to 12) is observed when cells are exposed to increasing concentrations of H_2O_2 for 1 hr. While exposure of ARPE-19 cells to H_2O_2 at 400 μM was sufficient to significantly enhance DJ-1 levels, a lower concentration (100 μM) was adequate to modulate DJ-1 levels in D407 cells. Quantitation of the intensity of immunoreactivity in blots from three independent experiments showed that DJ-1 increased 5.0 and 3.6 fold in ARPE-19 incubated with 400 and 600 μM H_2O_2 and up to 5.7 fold in D407 cells incubated with 200 μM H_2O_2 when compared with control cell RPE cultures (Fig. 2C). Similarly, both ARPE-19 (Fig. 2B, lanes 13 to 18) and D407 (Fig. 2b, lanes 19 to 24) also displayed a dose response when cells were exposed to increasing concentrations of H_2O_2 for 18 hrs. Quantitation of these blots showed that the intensity of DJ-1 immunoreactivity was 1.4, 1.3 and 1.8 fold higher in ARPE-19 incubated with 400 to 800 μM H_2O_2 and up to 1.6 fold greater in D407 cells incubated with 100 to 800 mM H_2O_2 when compared with control cell RPE cultures (Fig. 2D). Finally, ARPE-19 and D407 monolayers exposed to oxidative stress induced by incubation with increasing concentration of 4-hydroxynonenal (4-HNE), a lipid peroxidation product, for 12 (Figure S1) and 24 hrs (data not shown) also displayed increased DJ-1 immunoreactivity.

We also analyzed the distribution of DJ-1 in several RPE cell cultures plated on Transwell inserts non-incubated or incubated with 100 μM H_2O_2 overnight followed by immunofluorescence (Fig. 2E to J). Confocal microscopy *en face* examination of paraformaldehyde-fixed monolayers revealed a diffuse staining for DJ-1 consistent with a cytoplasmic localization in ARPE-19 (Fig. 2E), B6-RPE07 (Fig. 2G) and in mouse primary RPE (Fig. 2I) monolayers under baseline conditions. Oxidative stress induced by H_2O_2 leads to a visible increase in immunocytochemical staining for DJ-1 (Fig. 2F, H, J). In addition, to this visible increase in DJ-1 staining, an induced intracellular redistribution of DJ-1 to an aggregated, perinuclear localization was also observed (Fig. 2F, H,

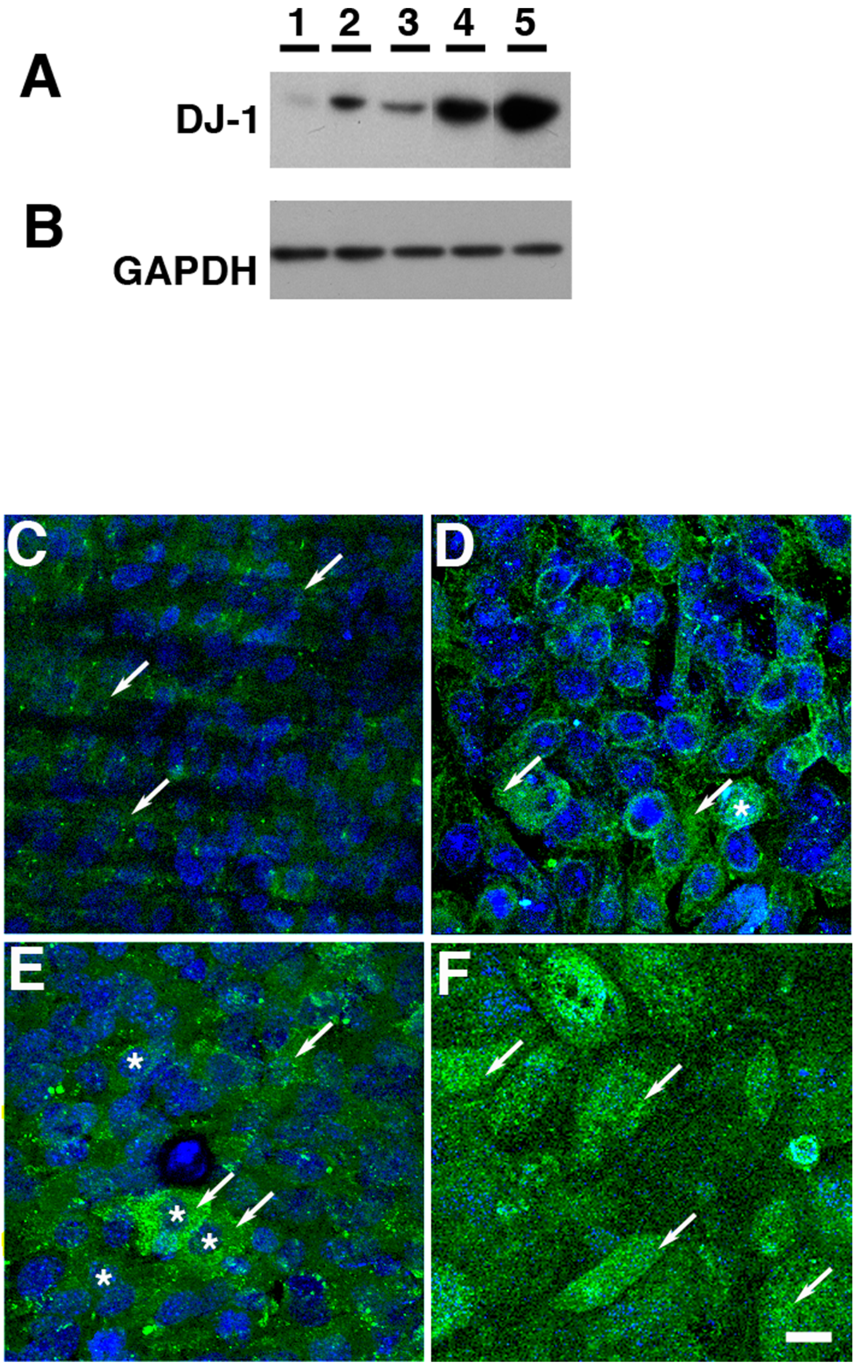

Figure 1. DJ-1 is present in RPE cell cultures. Lysates of the human RPE cell lines ARPE-19 (A, lane 1) and D407 (A, lane 2), the mouse RPE cell line B6-RPE07 (A, lane 3), mouse primary RPE (A, lane 4) and mouse brain lysates (A, lane 5) were harvested and analyzed by immunoblot assay with DJ-1 antibody. The DJ-1 signal varied in intensity in each RPE cell culture. Each lane contained 20 µg of protein. Protein loads were confirmed in replicate blots probed with GAPDH (B). Alternatively, monolayers of ARPE-19 (C), D407 (D), B6-RPE07 (E) and mouse primary RPE (F) were fixed with paraformaldehyde before extraction with Triton X-100 and labeling with DJ-1 antibodies. Cell nuclei were labeled with TO-PRO-3. DJ-1 labeling was diffusely distributed in the cytoplasm (arrows) and in the nuclei (*) of some of the cells (C-F, arrows). Scale bar = 20 µm.
doi:10.1371/journal.pone.0067983.g001

J). Our results suggested that DJ-1 immunoreactivity is increased and redistributed within RPE cells subjected to oxidative stress.

DJ-1 Localization in RPE Cells Subjected to Oxidative Stress

Further experiments were carried out in several RPE cultures plated on Transwell inserts non-incubated or incubated with 100 µM H_2O_2 overnight followed by immunofluorescence to confirm the intracellular localization of DJ-1 upon oxidative injury

(Fig. 3). Confocal microscopy *en face* examination of paraformaldehyde-fixed monolayers again revealed a diffuse staining for DJ-1 consistent with a cytoplasmic localization in mouse primary RPE (Fig. 3A) and in ARPE-19 (Fig. 2G) monolayers. Monolayers were also labeled with antibodies specific to the mitochondrial marker OxPhos Complex IV subunit I (COX IV, Fig. 3B, E, H, K). Under baseline conditions, both RPE monolayers displayed no significant colocalization with the mitochondrial marker in overlaid images (Fig. 3C and I). Oxidative stress induced by H_2O_2 lead to a visible increase in immunocytochemical staining for DJ-1 (Fig. 3D and J). In addition, an intracellular redistribution of DJ-1 immunoreactivity leading to COX IV colocalization was also observed (Fig. 3F and L).

Additional experiments carried out in RPE cells plated on coverslips and incubated with the mitochondrial staining MitoTracker were also performed (Figure S2). B6-RPE07 monolayers were fixed and labeled for DJ-1 (Figure S2A and S2D) after loading with the mitochondrial staining MitoTracker (Figure S2B and S2E). Similar to results reported above with antibody labeling, DJ-1 showed little specific localization to mitochondria under baseline conditions (Figure S2A and S2C). However, treatment with H_2O_2 caused increased redistribution of DJ-1 to mitochondria as shown in the overlain images (Figure S2D and S2F, arrows). In addition, an increase in mitochondria labeling is also evident in the RPE cells subjected to oxidative stress. Together, our observations indicate that DJ-1 is redistributed to the mitochondria in RPE cells under oxidative stress.

Detection of DJ-1 Oxidation at Cysteine 106 (oxDJ-1) in Cells Subjected to Oxidative Stress

DJ-1 has three cysteine (C) residues at amino acids 46, 53 and 106. C106 in DJ-1 is the first to become oxidized by the addition of cysteine sulfinic acid (C-SO_2H) and then C46 and C53 become oxidized upon oxidative stress, resulting in scavenging of reactive oxidative species (ROS) and enhancing DJ-1 association with mitochondria [23,47,48]. To check for alterations in DJ-1 oxidative stress, ARPE-19 cultures at baseline and under oxidative stress conditions were reacted with an antibody generated against a synthetic peptide containing SO_3H at C106 of DJ-1 [49]. If RPE cells under stress generate DJ-1 oxidized at C106, it may be increased during this time. Immunoblots of lysates of ARPE-19 cells obtained from cultures subjected to oxidative stress induced by exposure to H_2O_2 for 1 hr (Fig. 4A) and 18 hrs (Fig. 4B) demonstrated a progressive increase in oxDJ-1 with a dose response increase in cultures under short-term oxidative tress (Fig. 4A). Interestingly, the oxDJ-1 detected mostly in cultures under oxidative stress, displayed a molecular weight ~110 kDa. We also tested ARPE-19 cultures for the expression of oxDJ-1 by immunofluorescence (Fig. 4C to E). Polarized monolayers plated on Transwells under baseline culture conditions failed to display labeling with the antibody to oxDJ-1 (Fig. 4C). However, several cells displayed staining with the antibody to oxDJ-1 when monolayers were incubated with H_2O_2 for 1 h (Fig. 4D) and 18 hrs (Fig. 4E). The large increase of oxDJ-1 content when RPE cultures were under oxidative stress suggests that this DJ-1 in RPE indeed undergoes oxidation under oxidative stress.

Regulation and Distribution of DJ-1 Oxidation at C Residues in Cells Subjected to Oxidative Stress

The above data suggested that oxidation of DJ-1 C residues is correlated to the presence of oxidative stress. To further understand the function of DJ-1 oxidation RPE cultures were infected with adenoviruses carrying full-length human DJ-1 cDNA (hDJ-1 Ad) and a mutant construct, which has the C residues at amino acids 46, 53 and 106 mutated to serine (S), (C to S Ad) prior to stress experiments followed by evaluation of content and localization of DJ-1 in RPE cultures (Fig. 5). Immunoblots of lysates revealed that ARPE-19 cultures transduced with the hDJ-1 (Fig. 5A, lane 3) and with the C to S Ad constructs (Fig. 5, lane 2) displayed significant increased immunoreactivity of DJ-1 when compared to ARPE-19 control cultures (Fig. 5A, lane 1) and normalized to the levels of GAPDH (Fig. 5B) under baseline conditions. Quantification of these lysates demonstrated a 1.6 and 1.8 fold increase in the content of DJ-1 in ARPE-19 transduced with the C to S and hDJ-1 Ad, respectively (Fig. 5C), when comparing DJ-1 immunoreactivity to the one of ARPE-19 with normal levels of DJ-1. In addition, a significant increase in the immunoreactivity of DJ-1 was observed when ARPE-19 cultures were subjected to oxidative stress induced by H_2O_2 (Fig. 5A, +H_2O_2). Quantification of the immunoblots of these lysates demonstrated a 2.8, 1.4 and 4.8 fold increase in the immunoreactivity of DJ-1 in ARPE-19 cells and in ARPE-19 cells transduced with the C to S and hDJ-1 Ad, respectively (Fig. 5C), when comparing signal intensities to the one of ARPE-19 DJ-1 levels in baseline culture conditions.

To compare the distribution of endogenous and exogenous DJ-1 under baseline and oxidative stress conditions, ARPE-19 cells and ARPE-19 cells transducing exogenous DJ-1 were fixed and labeled for the distribution of DJ-1 and mitochondria (Fig. 5D to I). Double labeling cells on Transwells with antibodies to DJ-1 (green) and the mitochondria protein COX IV (red) showed little colocalization of DJ-1 with COX IV in control ARPE-19 cultures (Fig. 5D) and in ARPE-19 overexpressing full-length DJ-1 (Fig. 5E) and the C to S mutant DJ-1 (Fig. 5F) under baseline culture conditions. However, a significant colocalization of DJ-1 with COX IV could be observed in ARPE-19 cells (Fig. 5G and inset) and ARPE-19 cultures overexpressing full-length DJ-1 (Fig. 5H and inset) when incubated with H_2O_2. Opposing, ARPE-19 cultures overexpressing the C to S mutant DJ displayed no colocalization between the distribution of DJ-1 and COX IV (Figure 5I and inset) under oxidative stress. Altogether, our results showed that DJ-1 C residues are important for their increased response and redistribution to the mitochondria in cells subjected to oxidative stress.

Functional Role of DJ-1 C Oxidation in Cells Subjected to Oxidative Stress

To determine whether DJ-1 oxidation at its C residues could protect RPE cells against oxidative stress, we subjected ARPE-19 cells and ARPE-19 cells overexpressing full-length and C to S mutant DJ-1 to oxidative stress induced by H_2O_2 and labeling of ROS generation through to incubation with CM-H_2DCFDA (Fig. 6). No ROS were observed in ARPE-19 cells under normal culture conditions (Fig. 6A). However, significant intracellular ROS generation was observed when ARPE-19 (Fig. 6B) and ARPE-19 monolayers overexpressing the C to S mutant DJ-1 (Fig. 6C) were subjected to oxidative stress. Strikingly, no ROS was generated when ARPE-19 monolayers were overexpressing the full length DJ-1 (Fig. 6D). An inverse dose response of ARPE-19 monolayers overexpressing full length DJ-1 was observed when cells were infected with decreasing concentrations of hDJ-1 adenovirus (Fig. 6E and F), suggesting a gene-dosage effect. These results suggested that DJ-1 C oxidation is necessary for DJ-1 to protect RPE cells under oxidative stress from the intracellular generation of ROS.

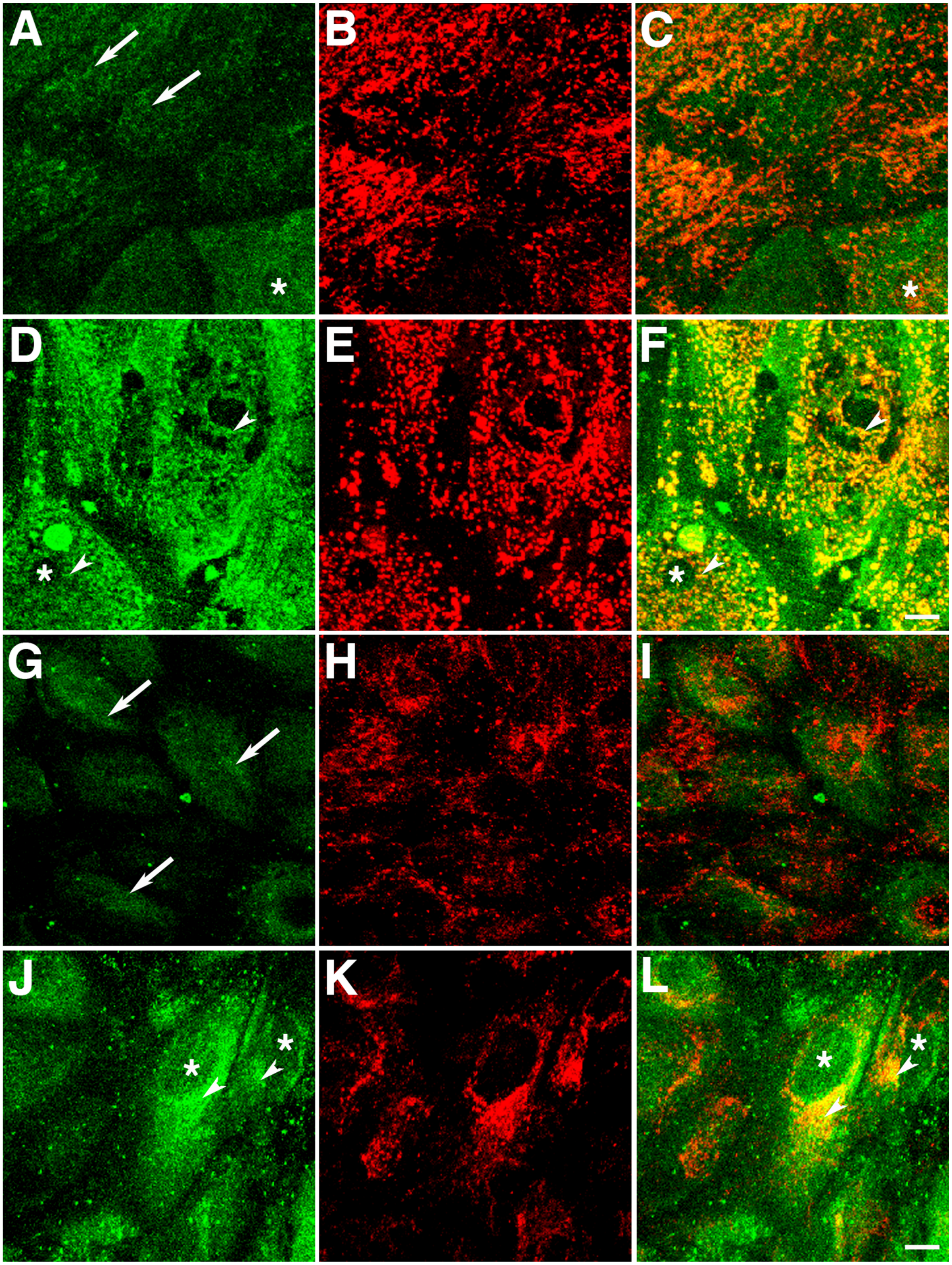

Figure 3. Oxidative stress-dependent translocation of DJ-1 into mitochondria. Representative confocal micrographs of mouse primary RPE (A–F) and ARPE-19 (G–L) monolayers plated on Transwells® and labeled with antibodies to DJ-1 (A, D, G, J) and COX IV (B, E, H, K). Under baseline conditions, there is very little colocalization between DJ-1 and COX IV, as observed in overlaid images (C, I) for both RPE cultures; DJ-1 is mostly distributed trough the cytoplasm (arrows) and to the nuclei (*) of some cells. Upon oxidative stress induced by incubation with 400 µM H_2O_2 for 1 hr, DJ-1 staining is increased both in the mouse primary (D) and ARPE-19 (J) cultures. In cultures treated with H_2O_2 some DJ-1 re-distributed to mitochondria (arrowheads) and displayed significant colocalization with COX IV in overlaid images (F, L). Scale bar = 10 µm.
doi:10.1371/journal.pone.0067983.g003

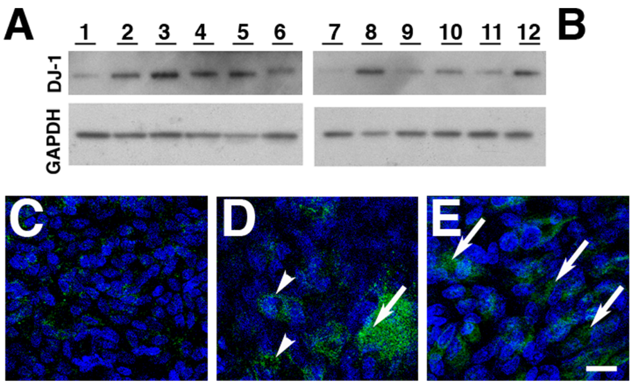

Figure 4. Presence of oxDJ-1 in RPE cells subjected to oxidative stress. ARPE-19 monolayers were treated with increasing concentrations (0 to 800 μM) of H_2O_2 for 1 hr (A) and 18 hs (B), harvested, and analyzed by immunoblot assay with oxDJ-1 antibody (upper panel). Protein loadings were confirmed in replicate blots probed with GAPDH (lower panel). Each lane contained 20 μg of protein. A dose response is observed when cells are exposed to increasing concentrations of H_2O_2 for 1 h (A, lanes 1 to 6) and 18 hrs (B, lanes 7 to 12). Confocal immunofluorescence staining of baseline ARPE-19 cultures (C) fixed before extraction with Triton X-100 and labeling with oxDJ-1 antibodies revealed absence of oxDJ-1. However, oxDJ-1 is observed in the cytoplasm (arrows) and perinuclear area (arrowheads) of RPE cells exposure to 400 μM H_2O_2 for 1 h (D) and 18 hrs (E). Cell nuclei were labeled with TO-PRO-3. Scale bar = 20 μm.
doi:10.1371/journal.pone.0067983.g004

Detection of DJ-1 in Human RPE Cells Isolated from the Eyes from AMD and non-AMD Donors

To better understand the significance of our RPE culture findings, DJ-1 presence in RPE lysates isolated from human donor eyes was carried out. DJ-1 presence was also probed in RPE cells isolated from donors with AMD, a blinding disease affecting the elderly, with an established contribution of oxidative stress to the pathogenesis of the disease (Fig. 7). DJ-1 was detected and highly expressed in the RPE lysates from AMD donors (Fig. 7A, lanes 6 to 10) when compared to the RPE lysates from non-AMD donors (Fig. 7A, lanes 1 to 5). DJ-1 immunoreactivity was normalized to the GAPDH content of the samples (Fig. 7C). Quantitation of these blots showed that DJ-1 immunoreactivity was increased ~2.5 fold in RPE isolated from AMD donors when compared with RPE isolated from non-AMD donors (Fig. 7D).

Our *in vitro* data showed that DJ-1 is oxidized at C106 when the cells are exposed to oxidative stress. To check if oxDJ-1 is present in RPE cells *in vivo*, RPE lysates from AMD and non-AMD donors were probed for this modification with a specific antibody (Fig. 7B). oxDJ-1 was present in the RPE lysates from AMD donors at higher levels (Fig. 7B, lanes 6 to 10) when compared to the RPE lysates from non-AMD donors (Fig. 7B, lanes 1 to 5). DJ-1 immunoreactivity was normalized to the GAPDH content of the samples (Fig. 7C). Quantitation of these blots showed that oxDJ-1 immunoreactivity was ~6 fold higher in RPE isolated from AMD donors when compared with RPE isolated from non-AMD donors (Fig. 7D).

We next immunohistologically examined the location of DJ-1 in RPE from non-AMD and AMD donor eyes with geographic atrophy (Fig. 7E to L) and isolated BM/choroid from the perimacula of AMD donors (Fig. 7M to P). Immunostaining of DJ-1 was detected mostly in the RPE nuclei (arrowheads) but also in the cytoplasm of non-AMD donors (Fig. 7F and H, arrows). Significantly more DJ-1 was detected through out the cytoplasm of RPE cells from two different AMD donors wit geographic atrophy (Fig. 7J and L, arrows). Interestingly, DJ-1 presence in RPE cells became less intense at distances away from the region of RPE atrophy (Figure S3). In addition, in BM/choroid isolated from two different AMD donors and drusen (insoluble aggregates localized underneath the RPE embodying the hallmark lesions of the disease, asterisks), in these samples were also specifically labeled with the DJ-1 antibody (Fig. 7N and P). In contrast, no significant DJ-1 labeling was observed when sections were reacted with the DJ-1 antibody, pre-absorbed with lysates from cells overexpressing exogenous DJ-1 (Fig. 7E, 7G, 7I, 7K, 7M and 7O). Our results showed that higher levels of DJ-1 and oxDJ-1 are present in RPE cells from AMD donors. In addition, we also reported that DJ-1 is also present in the hallmark lesion of this disease.

Discussion

Previous reports showed that DJ-1 is ubiquitously expressed in numerous tissues including the pancreas, kidney, skeletal muscle, liver, placenta, heart and brain, with high expression in astrocytes of the frontal cortex and substantia nigra [9]. We recently reported the identification of DJ-1 peptides in both young and aged RPE lysates using proteomics together with confirmation of the localization of DJ-1 in RPE cells in rat retinas using immunohistochemistry [44]. Here, we described DJ-1 increased expression and redistribution to mitochondria of RPE cells under oxidative stress. In addition, we report that overexpression of full-length DJ-1, prior to exposure to oxidative stress, led to significant decrease in the generation of ROS. Most importantly, increased DJ-1 and oxDJ-1 were detected by Western blot in human RPE lysates from AMD donors. Finally, immunohistology detected DJ-1in isolated human BM/choroid and in drusen from AMD eyes.

Results reported here in RPE cultures under baseline and oxidative stress conditions are in agreement with previous reporting of the subcellular distribution of DJ-1. At the subcellular level, under baseline conditions, DJ-1 is found mostly in the cytoplasm [32] and to a lesser extent in the mitochondria [23] and nucleus [50]. Under conditions of oxidative stress, more DJ-1 redistributes to mitochondria and later to the nucleus, and this correlates with the ability of DJ-1 to confer neuroprotection [23].

Our results reporting increased levels of DJ-1 in RPE cells subjected to oxidative stress induced by incubation with H_2O_2 and 4-HNE suggest that DJ-1 functions as a sensor of cellular redox homeostasis, which reacts to oxidative stress by increasing DJ-1 content. Similar data was previously reported when experiments were carried out in other cell types under oxidative stress induced by several agents [30,51,52,53,54,55,56,57].

DJ-1 has three C at amino acid numbers 53, 46 and 106, which can become oxidized upon oxidative stress and oxidation of C106 (oxDJ-1) is essential for DJ-1 to exert its full activities. In the present study we report the presence of oxDJ-1 in RPE cells in response to oxidative stress using antibodies that specifically recognize DJ-1 oxidized at C106 (oxDJ-1). Similar results have also been previously reported in other cells types and PD animal models [18,47,58,59,60]. DJ-1 has four Met (M) residues, which are also susceptible to oxidation in addition to the three C residues already cited. The oxidation of the M to the sulfoxide (1 O), and C to the sulfenic acid (1 O), sulfinic acid (2 Os) or sulfonic acid (3 Os) is expected to lead to increases in the number of O atoms in the protein ranging from four, if all four M residues are oxidized, and nine if all three C residues are fully oxidized, for a potential total of 13 additional oxygen atoms [18]. Interestingly, the molecular weight of the protein detected in RPE cells incubated with H_2O_2 displayed a higher molecular weight than the reported DJ-1 molecular weight (~25 kDa). The meaning of this observation is

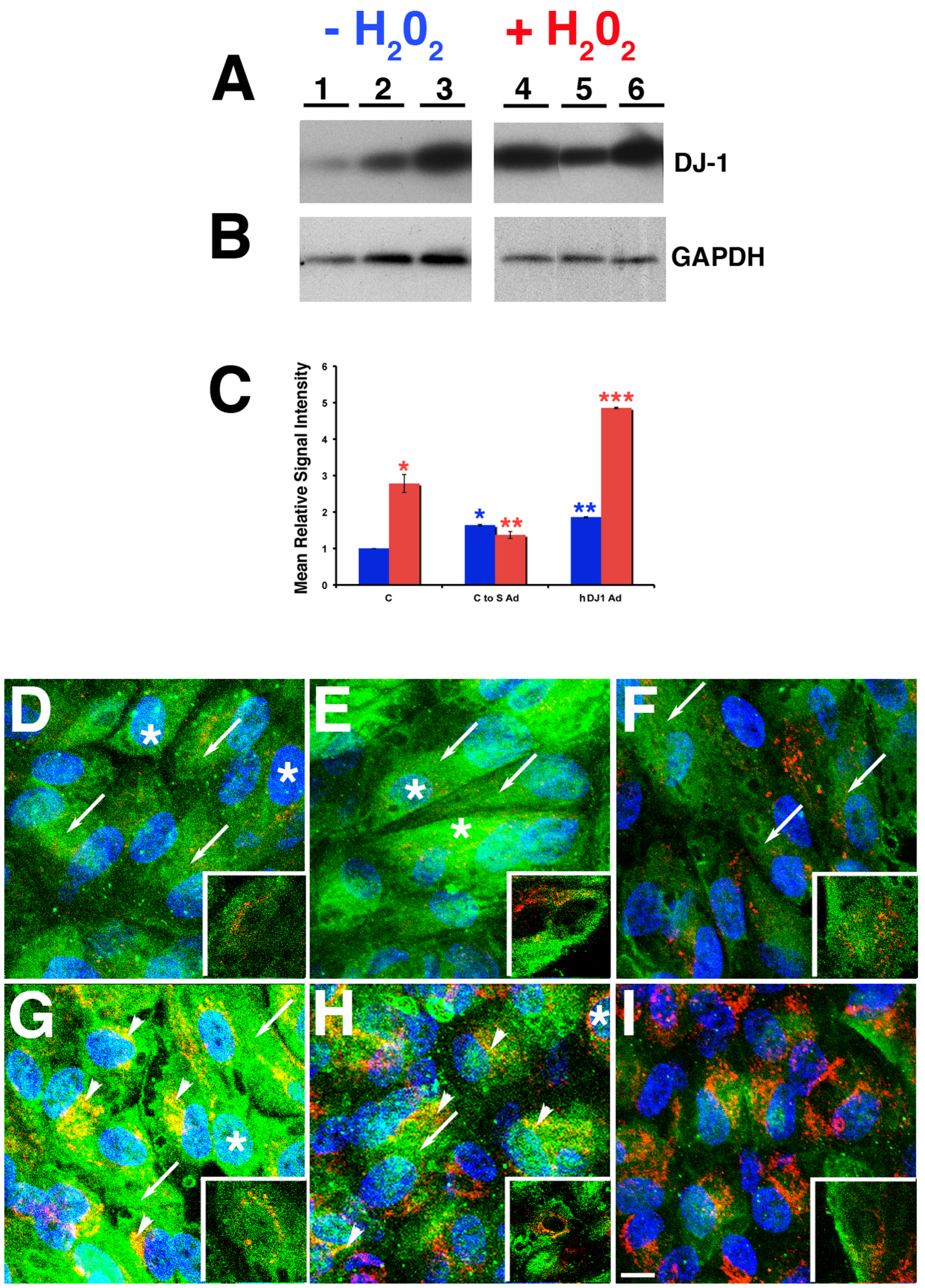

Figure 5. Overexpression of DJ-1 full-length lead to increased levels and redistribution in RPE cells upon oxidative stress. ARPE-19 monolayers were transduced with high titer (5×10^6 pfu) adenovirus carrying full length human DJ-1 (hDJ-1 Ad) or adenovirus carrying DJ-1 C to S mutant (C to S Ad). Forty-eight hours later cultures were treated (A, lanes 4 to 6) or not (A, lanes 1 to 3) with 400 µM H_2O_2, harvested, and analyzed by immunoblot assay with DJ-1 antibody (A). Each lane contained 20 µg of protein. Protein loads were confirmed in replicate blots probed with GAPDH (B). Immunoblots of lysates revealed that ARPE-19 cultures transduced with the hDJ-1 (A, lane 3) and with the C to S Ad constructs (A, lane 2) displayed significant increased immunoreactivity of DJ-1 when compared to ARPE-19 control cultures (A, lane 1) under baseline conditions. Quantification of immunoblots of these cultures demonstrated a 1.6 and 1.8 fold increase in the expression levels of DJ-1 in ARPE-19 transduced with the C to S and hDJ-1 Ad, respectively (C, blue columns). Blue columns = baseline ARPE-19 cultures; Red columns = ARPE-19 incubated with 400 µM H_2O_2 for 1 hr. Data expressed as mean relative signal intensity ± SEM ($n = 3$). Asterisks denote statistical significance compared with control ARPE-19 untreated cells (*$p = 0.0007$ in ARPE-19 transduced with C to S Ad and **$p < 0.0001$ ARPE-19 transduced with hDJ-1 Ad). In addition, a significant increase in the immunoreactivity of DJ-1 was observed when ARPE-19 cultures (A, lane 4) and ARPE-19 overexpressing the hDJ-1 Ad were subjected

to oxidative stress (A, lane 6). Quantification of the immunoblots of these lysates demonstrated a 2.8, 1.4 and 4.8 fold increase in the immunoreactivity of DJ-1 in ARPE-19 cells and in ARPE-19 cells transduced with the C to S and hDJ-1 Ad, respectively (C, red columns), when comparing signal intensities to the one of ARPE-19 not overexpressing exogenous DJ-1 in baseline culture conditions. These differences were statistically significant (*p = 0.0020 in the ARPE-19, **p = 0.0177 in ARPE-19 transduced with C to S Ad and ***p<0.0001 ARPE-19 transduced with hDJ-1 Ad). **D–I:** Confocal microscopy of ARPE-19 monolayers fixed, permeabilized with a buffer containing Triton X-100, and processed for indirect immunofluorescence with DJ-1 (green) and COX IV (red) antibodies. Cell nuclei were labeled with TO-PRO-3. Under baseline conditions there is very little colocalization between DJ-1 and COX IV in control ARPE-19 (D) and in ARPE-19 transduced with hDJ-1 Ad (E) and C to S Ad (F); DJ-1 is distributed in the cytoplasm (arrows) and nuclei (*) of cells. Upon oxidative stress induced by incubation with 400 μM H_2O_2 for 1 h, DJ-1 staining redistributed intracellularly to mitochondria (arrowheads) and colocalized with COX IV both in ARPE-19 (G) and ARPE-19 transduced with hDJ-1 Ad (H) but not in ARPE-19 cultures transduced with the C to S Ad (I). Insets: close up images of the DJ-1 and COX IV labeling. Scale bar = 10 μm.
doi:10.1371/journal.pone.0067983.g005

not fully known but it may be related to the generation of several DJ-1 isoforms resulting from the oxidation of the M and C residues [18,54,61].

The importance of DJ-1 C oxidation is also highlighted in RPE cells overexpressing C to S DJ-1 mutant that are exposed to oxidative stress, which are unable to increase DJ-1 levels and redistribute DJ-1 intracellularly to mitochondria. Our results, together with previous reports, suggests that DJ-1 C oxidation is important for protein stabilization [61,62,63,64] and mitochondria localization [23,52,65].

We demonstrated that overexpression of full-length but not the C to S mutant DJ-1 lead to significant decrease in the generation of intracellular ROS. The data suggests DJ-1 C oxidation – dependent elimination of ROS in cells under oxidative stress. Similar findings have been reported [52,57,66,67,68,69].

We demonstrate here for the first time that DJ-1 levels are increased in RPE lysates from AMD donors and that DJ-1 immunolocalization in RPE is also increased in AMD donors displaying geographic atrophy. AMD is a multi-factorial disease with known established risk factors including age, cigarette smoking, family history, gender, high blood pressure, high fat diet and race [70,71,72]. However, our understanding of the detailed molecular mechanisms of the development of AMD still remains limited, and to date, there is no proficient cure or preventive treatment. Oxidative stress affecting the physiological function and leading to the death of the RPE cells is a major factor contributing to the pathogenesis of AMD [6,7]. Therefore, limiting RPE oxidative stress may represent an effective way to slow or possibly reverse vision loss of patients due to diseases such as AMD. Indeed, several *in vitro* studies have shown that oxidative stress-related RPE cell death and dysfunction was improved when the

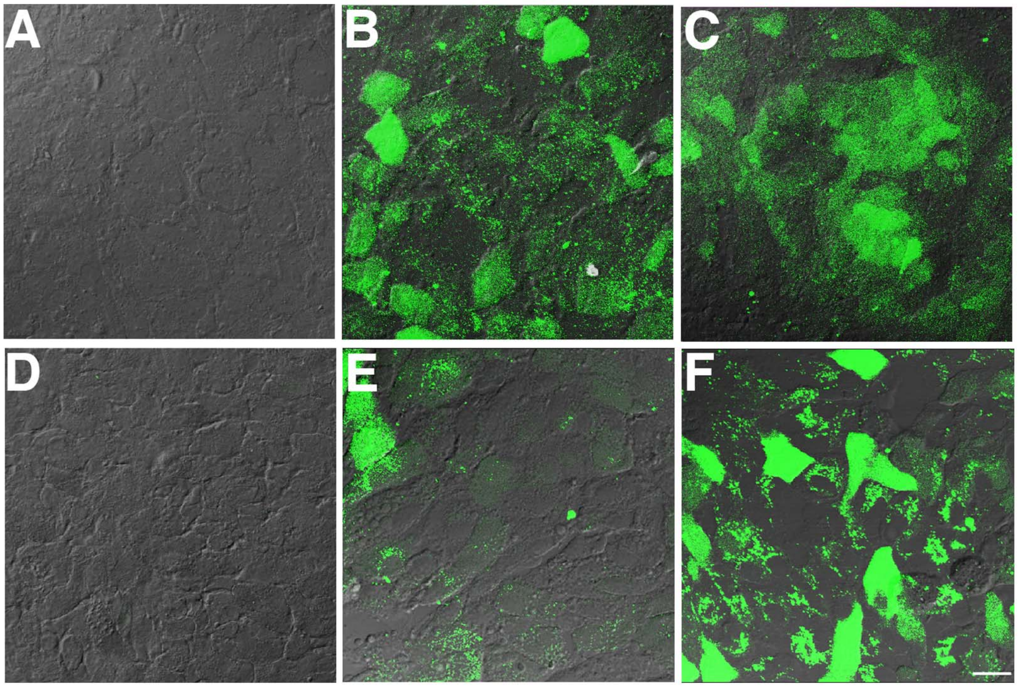

Figure 6. Overexpression of DJ-1 full length prior to oxidative stress leads to decreased levels of H_2O_2-induced ROS generation by RPE cells. ARPE-19 control cultures (A, B) and ARPE-19 transduced with adenovirus carrying full length human DJ-1 (hDJ-1 Ad) or adenovirus carrying DJ-1 C to S mutant (C to S Ad). Forty-eight hours following infection with adenovirus, cultures were treated with 800 μM H_2O_2 for 1 h followed by incubation with CM-H_2DCFDA and the fluorescence intensity of oxidized DCF (green) was then visualized by confocal microscopy. Cellular images were obtained by difference interference contrast (DIC) and overlaid on the fluorescence image of generated ROS. Images indicated that no ROS generation is observed in RPE cells at basal conditions (A) while upon oxidative stress incubation, appreciable ROS generation throughout the entire cell body of cells is observed (B). RPE cultures transduced with the high titer (5×10^6 pfu) of the C to S Ad displayed appreciable ROS generation when cells were exposed to oxidative stress (C). Markedly, RPE cultures transduced with the high titer of the hDJ-1 Ad (5×10^6 pfu) did not display ROS generation when exposed to oxidative stress (D). However, ROS generation was increasingly observed when RPE cultures were infected with increasing dilutions of hDJ-1 Ad, such as 4×10^6 pfu (E) and 3×10^6 pfu (F). Scale bar = 20 μm.
doi:10.1371/journal.pone.0067983.g006

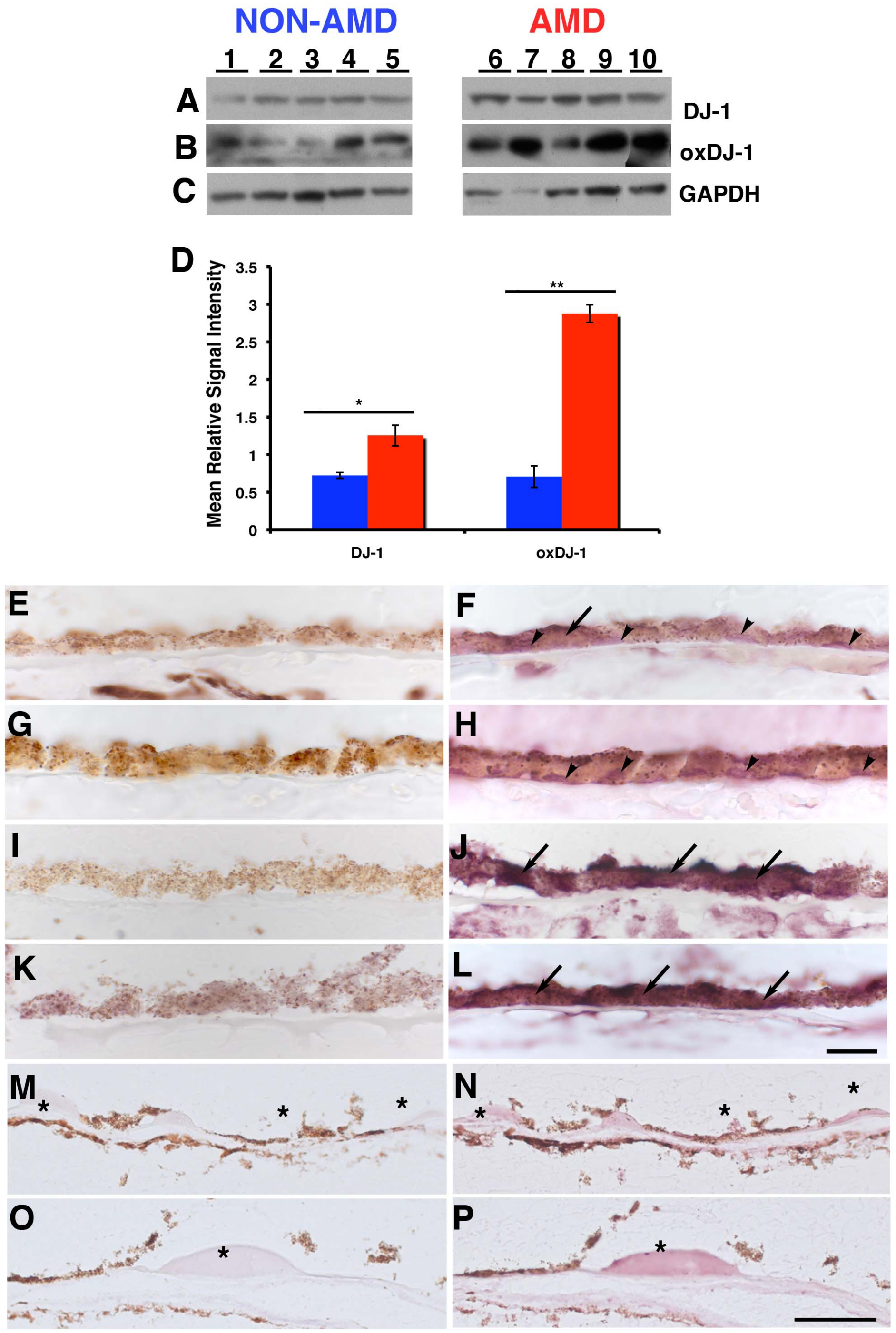

Figure 7. Increased levels of DJ-1 and oxDJ-1 in RPE lysates and tissue from AMD donors. Lysates of human RPE isolated from non-AMD (A to C lanes 1 to 5) and AMD donors (A to C, lanes 6 to 10) were harvested and analyzed by immunoblot assay with DJ-1 antibody (A) and oxDJ-1 antibody (B). Each lane contained 20 µg of protein. Protein loads were confirmed in replicate blots probed with GAPDH (C). Immunoblots of lysates revealed that AMD RPE displayed significant increased immunoreactivity of both DJ-1 (A) and oxDJ-1 (B) when compared to non-AMD RPE lysates. Quantification of immunoblots demonstrated a 1.7 and 4 fold increase in the expression levels of DJ-1 and oxDJ-1, respectively (D). Blue columns = non-AMD; Red columns = AMD. Data is expressed as mean relative signal intensity ± SEM (n = 8). Asterisks denote statistical significance compared with non-AMD RPE (*p = p = 0.0098 for DJ-1 and **p = 0.0058 for oxDJ-1). Alternatively, cryosections of different non-AMD (E–H) and AMD (I to L) donors with geographic atrophy, and isolated Bruch's membrane (BM) and choroid from two different AMD donors were (M to P) labeled with DJ-1 antibody. Negative control sections were reacted with DJ-1 antibody pre-absorbed with lysates of cells overexpressing DJ-1 and shoed no DJ-1 labeling (E, G, I, K, M, O). DJ-1 labeling was detected mostly in the RPE nuclei (arrowheads) but also in the cytoplasm of non-AMD donors (F and H, arrows). Significantly more DJ-1 was detected all over the cytoplasm of RPE cells from two different AMD donors (J and L, arrows), while DJ-1 was diffusely distributed in isolated BM and in drusen (E, G, asterisks). Scale bars (E to L) = 10 µm; (M to P) = 50 µm.
doi:10.1371/journal.pone.0067983.g007

cells were treated with antioxidants [73,74,75,76]. Furthermore, a significant reduction toward retinal degeneration was reported in clinical trials involving AMD patients ingesting antioxidants such as lutein, zeaxanthin, zinc, vitamin C, and vitamin E [77,78]. It is likely that the increased levels of DJ-1 in RPE lysates from AMD donors is related to increased oxidative stress in these RPE cells *in vivo*. Increased levels of DJ-1 were also reported in the brains of PD and Alzheimer's disease (AD) patients [27].

The increased presence of oxDJ-1 was reported in corneal buttons from Fuchs endothelial corneal dystrophy patients [56]. In addition, the presence of several oxidized DJ-1 isoforms have been found in patients with PD [79] and in patients with AD [27]. These data suggest that the DJ-1 oxidation status modulates its functions and that deregulation of DJ-1 oxidation may lead to the onset of diseases such as AMD [48]. In the present study we detected the increased presence of oxDJ-1 in RPE lysates from AMD donors using an antibody that detects oxidation at C106. Future experiments will be required to fully understand the significance of our finding.

A high number of mitochondria are present in the RPE due to its high metabolic activity [80]. During oxidative phosphorylation, the mitochondria produce the majority of the cellular energy in the form of ATP and also generate ROS as well [81,82]. A vicious circle of destruction takes place when ROS, including those produced in mitochondria by the electron transport chain, are generated leading to the preferred damage of mitochondrial genome (mtDNA). In turn, damaged mtDNA induces mitochondrial dysfunction with disturbance of oxidative phosphorylation and even higher production of ROS [2,83]. Therefore, it is not surprising that mitochondrial dysfunctions leading to increased ROS generation and mtDNA damage and specific hapogroups have been implicated in the pathophysiology of AMD [84,85,86,87,88,89,90,91,92]. We have shown here that an increased association of DJ-1 with mitochondria is observed in cells under oxidative stress. Therefore, it is likely that DJ-1 is "over-oxidized" in RPE cells from AMD patients failing to associate with the mitochondria and protect RPE cells from oxidative stress. Further experiments should concentrate on the analysis of DJ-1 intracellular distribution and oxidative isoforms in AMD and non-AMD RPE.

We reported here that DJ-1 was detected in Bruch's membrane and drusen isolated from two different AMD donors raising the question of how a cytoplasmic protein could be detected extracellularly. Previously, we [93,94] and other groups [95,96,97,98] reported that many of proteins found in drusen are normally found intracellularly in the RPE. These results suggest that RPE cells from AMD donors release intracellular proteins along their basal surface through a yet unknown mechanism where they become concentrated in drusen. DJ-1 is an intracellular protein. However, recently DJ-1 has emerged as a significant biomarker since its presence has been detected in the serum of gastric cancer [99], prostate carcinomas [100], pancreatic cancer [101], non-small cell lung cancer [102] and uveal melanoma [103] patients. Similarly, higher DJ-1 levels have been noted in nipple secretions from breast carcinoma patients [104]. Moreover, DJ-1 has been detected in cerebrospinal fluid from Parkinson's Disease patients [105,106,107] and urine from hepatocellular carcinoma [108] patients. Future studies will be needed to investigate how RPE cells release DJ-1 and to determine if it can be detected in the serum of AMD patients.

In summary, due to the evidence presented here connecting DJ-1 to protection against oxidative stress, it is conceivable that manipulation of DJ-1 function may be used to protect RPE cells from the oxidative stress implicated in AMD pathology.

Methods

Ethics Statement

The immunocytochemistry and Western analysis of human isolate RPE and BM/choroid is exempt of IRB approval since the human tissue was obtained and used after deceased.

All animal work was conducted in compliance with the Animal Welfare Act and Public Health Services policies, and under the oversight and approval of the Cleveland Clinic Institutional Animal Care and Use Committee (IACUC, protocol number ARC 2010-0136). All efforts were made to minimize animal suffering.

RPE Cell Cultures

Unless specified all media components were prepared in-house at the Cleveland Clinic cell culture lab core from commercially produced powders from Invitrogen, Sigma-Aldrich, and Caisson Labs.

The established cell line D407 [109], obtained as a gift from Dr. Richard Hunt (University of South Carolina School of Medicine, SC) was cultured at the temperature of 37°C as previously described [109]. B6-RPE07 mouse cells were cultured as previously described [45]. Briefly, cells were grown in Dulbecco's modified Eagle's medium (DMEM) supplemented with 3% heat-inactivated fetal bovine serum, glutamax (Gibco), non-essential amino acids, and penicillin/streptomycin. To promote differentiation, B6-RPE and D407 cells, which have a very high rate of growth, were transferred to and cultured in serum-free epithelial medium for two passages (Quantum 286 for epithelia cells; PAA Laboratories Ltd.) before plating on laminin-(BD Biosciences, San Jose, CA) and collagen-coated Transwell inserts (Corning, Corning, NY), respectively. The human RPE cell line ARPE-19 was obtained from American Type Culture Collection (Manassas, VA, USA). The cells were maintained in DMEM/F12 1:1 containing 10% FBS and 5.5 mmol D-glucose in a humidified incubator at 37°C in 5% CO2. The medium was changed every 3–4 days. Polarized ARPE-19 cells were plated and cultured for 3 weeks on collagen-coated Transwell inserts in 1% FBS medium before using in experiments. Primary mouse retinal pigment epithelium was isolated as previously described [110]. Briefly, eyes from ~2 week-old C57BL/6J mice were treated with 0.5 mg/ml bovine hyaluronidase (Sigma Chemical, St. Louis, MO) and 0.05 mg/ml of collagenase (Sigma-Aldrich) followed by and 0.1% trypsin (Difco-BD Biosciences, Sparks, MD) in buffer for 60 min each incubation to allow the mechanical separation of the neural retina and exposure of the RPE. Patches of RPE were peeled off manually from Bruch's membrane. To further dissociate the RPE patches, purified RPE cells were incubated with 0.05% trypsin/0.53 mM EDTA for 2 min at 37°C.

Human Eye Tissue

Donor eyes were obtained from the Cleveland Eye Bank or through the Foundation Fighting Blindness Eye (FFB) Donor Program (Columbia, MD). Tissue from 22 different donors were analyzed including 12 samples from non-AMD donors and 17 from AMD donors many of which, had previously been described [111]. The donor ages ranged between 35 and 91 years and the interval between time of death and tissue processing varied between 4 and 35.5 hours. Eye bank records accompanying the donor eyes indicated whether the donor had AMD or no known eye diseases.

Oxidative Stress Treatment

Monolayers were rinsed with warm PBS and incubated with PBS for 5 min at 37°C. Oxidative stress was induced by incubation with culture medium supplemented with 0–800 µM H_2O_2 for 1 and 24 hours. Alternatively, monolayers were stressed with 0–100 µM 4-HNE for 12 and 24 hours in SFM. Following this incubation, monolayers were washed with pre-warmed PBS and either fixed with 4% paraformaldehyde made in PBS and processed for immunofluorescence labeling or scraped and pelleted down to be processed for biochemistry analysis.

Adenovirus Infection

ARPE-19 cells were cultured as previously described. The replication-defective adenovirus vectors Ad5CMVPARK7 (for expression of human DJ-1 under control of a human cytomegalovirus [CMV] promoter) and AdCMVPARK7. C to S (for expression of human DJ-1 with the cysteine at residues 46, 53 and 106 mutated to serine) were prepared and titered by Welgen Inc. (Worcester, MA) using PARK7 human cDNA clone obtained from Origene (SC115623, Rockville, NY). To transduce cells, adenoviruses were mixed with transduction medium (20 mM Hepes-buffered DMEM containing 0.2% BSA) and incubated with cells for 2 h at a concentration of 5×10^6 plaque forming units (pfu)/cell unless specified in text. Cells on Transwells had viruses added to both apical and basal surface. After infection, transduction medium was replaced by normal culture medium and cells were returned to incubator. Two days after adenovirus transduction, cells were washed with pre-warmed PBS and either fixed with 4% paraformaldehyde made in PBS and processed for immunofluorescence or scraped and pelleted down to be processed for biochemistry analysis. Alternatively, cells were exposed to oxidative stress as described above and then processed for immunofluorescence or biochemistry analysis.

Cell Loading with Fluorescent Probe to Detect Reactive Oxygen Species (ROS) Production

ARPE-19 monolayers were plated on glass coverslips and cultured as described above. Monolayers infected or not with adenoviruses as described above, were subjected to oxidative stress as described above. A 10 µM stock solution of CM-H_2DCFDA (Molecular Probes, C6827) was prepared in DMSO immediately before use. Monolayers were loaded by a 15-min incubation with 10 µM DCFH-DA at room temperature in the dark. Following incubation, monolayers were washed with pre-warmed PBS at room temperature and carefully mounted immediately on the microscope slide in vectashield. Labeled cells were analyzed using a Leica laser scanning confocal microscope (TCS-SP2, Leica, Exton, PA). Cellular images were obtained by difference interference contrast (DIC).

Immunofluorescence of Cells

RPE monolayers on Transwells were fixed in 4% paraformaldehyde for 30 minutes at 4°C. Cells were blocked in PBS +1% BSA and incubated overnight at 4°C with poyclonal antibody directed against DJ-1 (NB300-270, 1:750; Novus), and DJ-1 oxidized at C106 (oxDJ-1, HCA024, 1:50; AbD serotec, Oxford, UK) and monoclonal antibody directed against OxPhos Complex IV subunit I (COX IV, 459600, 1:500; Invitrogen). Alexa Fluor488, Alexa Fluor594 and anti-Myc Tag Alexa Fluor488 secondary antibodies were added at room temperature for 1 hour (1:1000; Molecular Probes and 1:500; Millipore) and cell nuclei were labeled with TO-PRO®-3 iodide (1:5000; Molecular Probes). Monolayers were analyzed using a Leica laser scanning confocal microscope. A series of 1 µm *xy* (*en face*) sections were collected. Each individual *xy* image of the retinas stained represents a three-dimensional projection of the entire cryosection (sum of all images in the stack). Microscopic panels were composed using Adobe Photoshop CS3 (Adobe, San Jose, CA).

Immunohistology of Human Donor Tissue

To determine the localization of DJ-1 in RPE cells from non–AMD and AMD eyes and BM/choroid isolated from AMD donors, immunohistochemical assays were performed using cryosections in the peri-macular area. Isolated BM/choroid strips were isolated from the eyecups as previously described [94]. Eye pieces of retina-RPE-choroid were cut and fixed by immersion in 4% paraformaldehyde made in PBS overnight at 4°C, quenched with 50 mM NH4Cl made in PBS for 1 h at 4°C, infused successively with 10% and 20% sucrose made in the same buffer and with Tissue-Tek "4583" (Miles Inc., Elkhart, IN) as previously described [111]. Cryosections (8 µm) were cut on a cryostat HM 505E (Microm, Walldorf, Germany) equipped with a CryoJane Tape-Transfer system (Instrumedics, Inc., Hackensack, NJ, USA). Cryosections were washed, and processed for antigen retrieval in pre-heated Trilogy (Cell Marque, Rocklin, CA) by incubation for 30 min. in a steamer followed by transfer to room temperature for 20 min. to allow cryosections to cool down. Sections were blocked with PBS +1% BSA and probed with the DJ-1 antibody (TA301239, 1:750, Origene) overnight at 4°C followed by labeling with Vectastain Elite ABC reagent (Vector Laboratories, Inc., Burlingame, CA) according to the manufacture's directions. Negative controls were pre-absorbed overnight in the rotator with 5 µg of lysates of HEK293 cells overexpressing PARK7. Labeling was detected through incubation with ImmPACT VIP peroxidase substrate (Vector Laboratories) according to the manufacturer's instructions. Slides were mounted in cytoseal (Richard-Allan-Scientific, Kalamazoo, MI). The sections were examined with a Zeiss AxioImager.Z1 light microscope and the images were digitized using a Zeiss AxioCam MRc5 camera.

Western Blot Analysis

RPE cells were solubilized in RIPA buffer (0.1% SDS, 1% Triton X100, 1% deoxycholate, 0.15 M NaCl, 2 mM EDTA, 25 mM Tris pH 7.4) supplemented with a cocktail of protease and phosphatase inhibitors (Sigma Chemical Co., St. Louis, MO). Total RPE lysates (20 µg protein) were resolved by SDS-PAGE on 4–20% Novex®-Tris-Glycine gel (Invitrogen Corporation, Carlsbad, CA) and electro-transferred to Immobilon PVDF membranes (Millipore, Bedford, MA). Membranes were blocked with HyBLOCKER liquid blocking reagent (Denville Scientific Inc., Metuchen, NJ) for 30 min. and incubated overnight in the same solution with antibodies to DJ-1 (NB300-270, 1:2000; Novus), DJ-1 oxidized at C106 (oxDJ-1, HCA024, 1:200; AbD serotec), GAPDH (ab9484, 1:1000; Abcam). Protein detection was performed with secondary antibodies conjugated to peroxidase and visualized using ECL Plus Western Blotting detection reagent (GE Healthcare Bio-Sciences Corp, Piscataway, NJ). PVDF membranes were exposed to film. Films were scanned and figures were composed using Adobe Photoshop CS3. Signal intensities were quantified using ImageJ 1.43u (http://rsb.info.nih.gog/ij).

Supporting Information

Figure S1 Oxidative stress induced by 4-HNE increases DJ-1 levels in RPE cells. ARPE-19 and D407 monolayers were treated with increasing concentrations (0 to 100 µM) of 4-HNE for 12 hrs (A) harvested, and analyzed by immunoblot assay with DJ-1

antibody (upper panel). Each lane contained 20 μg of protein. Protein loadings were confirmed in replicate blots probed with GAPDH (lower panel). A dose response of ARPE-19 (A, lanes 1 to 6) and D407 (A, lanes 7 to 12) is observed when cells are exposed to increasing concentrations of 4-HNE for 12 hrs. Quantitation of these blots showed that DJ-1 immunoreactivity was 1.5 and 1.4 fold higher in ARPE-19 incubated with 5 and 10 μM HNE and up to 2.1 fold in D407 cells incubated with 25 μM HNE when compared with control cell RPE cultures (B). Plotted data represent the intensity values for each band normalized to GAPDH signal and compared to the intensity of the control, untreated cells (lanes 1, 7). Red columns = ARPE-19; blue columns = D407 cells. Data is expressed as mean relative signal intensity ± SEM (n = 3). Asterisks denote statistical significance compared with control untreated cells (*p = 0.0097 and **p<0.0001 in the ARPE-19 and *p = 0.0006, **p = 0.0499, ***p = 0.0020, ****p<0.0001 in D407 cells).
(TIF)

Figure S2 Oxidative stress-dependent translocation of DJ-1 into mitochondria. Representative confocal micrographs of B6-RPE07 monolayers plated on glass coverslips and labeled with antibodies to DJ-1 (A, D) and the mitochondrial staining MitoTracker (B, E). Cell nuclei were labeled with TO-PRO-3. Under baseline conditions, there is very little colocalization between DJ-1 and MitoTracker, as observed in overlaid images (C). Upon oxidative stress induced by incubation with 200 μM H_2O_2 for 18 hrs, the diffused cytoplasmic DJ-1 staining disappears. Moreover, in overlaid images a pronounced mitochondrial staining for DJ-1 is apparent when cells are exposed to oxidative stress (F, arrows). Scale bar = 20 μm.
(TIF)

Figure S3 Increased levels of DJ-1 in region of RPE atrophy in AMD donor. Cryosections of non-AMD (A) and AMD (B, C) donors with geographic atrophy were labeled with DJ-1 antibody. DJ-1 labeling was detected mostly in the RPE nuclei (arrowheads) but also in the cytoplasm (A, arrows) of non-AMD donors; labeling was also observed in the choriocapillaris (A, double arrowheads). Significantly more DJ-1 was detected all over the cytoplasm of RPE cells (B, arrows) and choriocapillaris (B, double arrowheads) from an AMD donor with geographic atrophy in the atrophic region; labeling was also significantly more intense in the choriocapillaris in this region. DJ-1 labeling of a druse (B, asterisks) is also observed. Interestingly, in this same AMD donor eye, at distances away from the region of RPE atrophy, DJ-1 immunoreactivity was similar to that observed in the RPE normal control eyes (C). Scale bars = 10 μm.
(TIF)

Acknowledgments

The authors thank Xiaoping Yang for technical assistance in the preparation of this paper.

Author Contributions

Conceived and designed the experiments: VLB. Performed the experiments: KGS MER VLB. Analyzed the data: VLB JGH. Contributed reagents/materials/analysis tools: VLB JGH. Wrote the paper: VLB.

References

1. Bok D (1993) The retinal pigment epithelium: a versatile partner in vision. J Cell Sci Suppl 17: 189–195.
2. Liang FQ, Godley BF (2003) Oxidative stress-induced mitochondrial DNA damage in human retinal pigment epithelial cells: a possible mechanism for RPE aging and age-related macular degeneration. Exp Eye Res 76: 397–403.
3. Tanito M, Brush RS, Elliott MH, Wicker LD, Henry KR, et al. (2009) High levels of retinal membrane docosahexaenoic acid increase susceptibility to stress-induced degeneration. J Lipid Res 50: 807–819.
4. Miceli MV, Liles MR, Newsome DA (1994) Evaluation of oxidative processes in human pigment epithelial cells associated with retinal outer segment phagocytosis. Exp Cell Res 214: 242–249.
5. Tate DJ, Jr., Miceli MV, Newsome DA (1995) Phagocytosis and H2O2 induce catalase and metallothionein gene expression in human retinal pigment epithelial cells. Invest Ophthalmol Vis Sci 36: 1271–1279.
6. Beatty S, Koh H, Phil M, Henson D, Boulton M (2000) The role of oxidative stress in the pathogenesis of age-related macular degeneration. Surv Ophthalmol 45: 115–134.
7. Drobek-Slowik M, Karczewicz D, Safranow K (2007) [The potential role of oxidative stress in the pathogenesis of the age-related macular degeneration (AMD)]. Postepy Hig Med Dosw (Online) 61: 28–37.
8. Bonifati V, Rizzu P, Squitieri F, Krieger E, Vanacore N, et al. (2003) DJ-1(PARK7), a novel gene for autosomal recessive, early onset parkinsonism. Neurol Sci 24: 159–160.
9. Nagakubo D, Taira T, Kitaura H, Ikeda M, Tamai K, et al. (1997) DJ-1, a novel oncogene which transforms mouse NIH3T3 cells in cooperation with ras. Biochem Biophys Res Commun 231: 509–513.
10. Hod Y (2004) Differential control of apoptosis by DJ-1 in prostate benign and cancer cells. J Cell Biochem 92: 1221–1233.
11. Gao X, Ning Y (2011) Cancer and Parkinson's disease: the odd couple. Drugs Today (Barc) 47: 215–222.
12. Hod Y, Pentyala SN, Whyard TC, El-Maghrabi MR (1999) Identification and characterization of a novel protein that regulates RNA-protein interaction. J Cell Biochem 72: 435–444.
13. Shinbo Y, Taira T, Niki T, Iguchi-Ariga SM, Ariga H (2005) DJ-1 restores p53 transcription activity inhibited by Topors/p53BP3. Int J Oncol 26: 641–648.
14. Zhong N, Kim CY, Rizzu P, Geula C, Porter DR, et al. (2006) DJ-1 transcriptionally up-regulates the human tyrosine hydroxylase by inhibiting the sumoylation of pyrimidine tract-binding protein-associated splicing factor. J Biol Chem 281: 20940–20948.
15. Clements CM, McNally RS, Conti BJ, Mak TW, Ting JP (2006) DJ-1, a cancer- and Parkinson's disease-associated protein, stabilizes the antioxidant transcriptional master regulator Nrf2. Proc Natl Acad Sci U S A 103: 15091–15096.
16. Takahashi K, Taira T, Niki T, Seino C, Iguchi-Ariga SM, et al. (2001) DJ-1 positively regulates the androgen receptor by impairing the binding of PIASx alpha to the receptor. J Biol Chem 276: 37556–37563.
17. Shendelman S, Jonason A, Martinat C, Leete T, Abeliovich A (2004) DJ-1 is a redox-dependent molecular chaperone that inhibits alpha-synuclein aggregate formation. PLoS Biol 2: e362.
18. Zhou W, Zhu M, Wilson MA, Petsko GA, Fink AL (2006) The oxidation state of DJ-1 regulates its chaperone activity toward alpha-synuclein. J Mol Biol 356: 1036–1048.
19. Yoshida K, Sato Y, Yoshiike M, Nozawa S, Ariga H, et al. (2003) Immunocytochemical localization of DJ-1 in human male reproductive tissue. Mol Reprod Dev 66: 391–397.
20. Mitsumoto A, Nakagawa Y (2001) DJ-1 is an indicator for endogenous reactive oxygen species elicited by endotoxin. Free Radic Res 35: 885–893.
21. Taira T, Saito Y, Niki T, Iguchi-Ariga SM, Takahashi K, et al. (2004) DJ-1 has a role in antioxidative stress to prevent cell death. EMBO Rep 5: 213–218.
22. Martinat C, Shendelman S, Jonason A, Leete T, Beal MF, et al. (2004) Sensitivity to oxidative stress in DJ-1-deficient dopamine neurons: an ES-derived cell model of primary Parkinsonism. PLoS Biol 2: e327.
23. Canet-Aviles RM, Wilson MA, Miller DW, Ahmad R, McLendon C, et al. (2004) The Parkinson's disease protein DJ-1 is neuroprotective due to cysteine-sulfinic acid-driven mitochondrial localization. Proc Natl Acad Sci U S A 101: 9103–9108.
24. Takahashi-Niki K, Niki T, Taira T, Iguchi-Ariga SM, Ariga H (2004) Reduced anti-oxidative stress activities of DJ-1 mutants found in Parkinson's disease patients. Biochem Biophys Res Commun 320: 389–397.
25. Kim RH, Smith PD, Aleyasin H, Hayley S, Mount MP, et al. (2005) Hypersensitivity of DJ-1-deficient mice to 1-methyl-4-phenyl-1,2,3,6-tetrahydropyrindine (MPTP) and oxidative stress. Proc Natl Acad Sci U S A 102: 5215–5220.
26. Park J, Kim SY, Cha GH, Lee SB, Kim S, et al. (2005) Drosophila DJ-1 mutants show oxidative stress-sensitive locomotive dysfunction. Gene 361: 133–139.
27. Choi J, Sullards MC, Olzmann JA, Rees HD, Weintraub ST, et al. (2006) Oxidative damage of DJ-1 is linked to sporadic Parkinson and Alzheimer diseases. J Biol Chem 281: 10816–10824.
28. Meulener MC, Xu K, Thomson L, Ischiropoulos H, Bonini NM (2006) Mutational analysis of DJ-1 in Drosophila implicates functional inactivation by oxidative damage and aging. Proc Natl Acad Sci U S A 103: 12517–12522.

29. Kahle PJ, Waak J, Gasser T (2009) DJ-1 and prevention of oxidative stress in Parkinson's disease and other age-related disorders. Free Radic Biol Med 47: 1354–1361.
30. Mitsumoto A, Nakagawa Y, Takeuchi A, Okawa K, Iwamatsu A, et al. (2001) Oxidized forms of peroxiredoxins and DJ-1 on two-dimensional gels increased in response to sublethal levels of paraquat. Free Radic Res 35: 301–310.
31. Zhou W, Freed CR (2005) DJ-1 up-regulates glutathione synthesis during oxidative stress and inhibits A53T alpha-synuclein toxicity. J Biol Chem 280: 43150–43158.
32. Bonifati V, Rizzu P, van Baren MJ, Schaap O, Breedveld GJ, et al. (2003) Mutations in the DJ-1 gene associated with autosomal recessive early-onset parkinsonism. Science 299: 256–259.
33. Junn E, Taniguchi H, Jeong BS, Zhao X, Ichijo H, et al. (2005) Interaction of DJ-1 with Daxx inhibits apoptosis signal-regulating kinase 1 activity and cell death. Proc Natl Acad Sci U S A 102: 9691–9696.
34. Xu J, Zhong N, Wang H, Elias JE, Kim CY, et al. (2005) The Parkinson's disease-associated DJ-1 protein is a transcriptional co-activator that protects against neuronal apoptosis. Hum Mol Genet 14: 1231–1241.
35. Malhotra D, Thimmulappa R, Navas-Acien A, Sandford A, Elliott M, et al. (2008) Decline in NRF2-regulated antioxidants in chronic obstructive pulmonary disease lungs due to loss of its positive regulator, DJ-1. Am J Respir Crit Care Med 178: 592–604.
36. Yokota T, Sugawara K, Ito K, Takahashi R, Ariga H, et al. (2003) Down regulation of DJ-1 enhances cell death by oxidative stress, ER stress, and proteasome inhibition. Biochem Biophys Res Commun 312: 1342–1348.
37. van der Brug MP, Blackinton J, Chandran J, Hao LY, Lal A, et al. (2008) RNA binding activity of the recessive parkinsonism protein DJ-1 supports involvement in multiple cellular pathways. Proc Natl Acad Sci U S A 105: 10244–10249.
38. Ottolini D, Cali T, Negro A, Brini M (2013) The Parkinson disease-related protein DJ-1 counteracts mitochondrial impairment induced by the tumour suppressor protein p53 by enhancing endoplasmic reticulum-mitochondria tethering. Hum Mol Genet.
39. Larsen NJ, Ambrosi G, Mullett SJ, Berman SB, Hinkle DA (2011) DJ-1 knock-down impairs astrocyte mitochondrial function. Neuroscience 196: 251–264.
40. Mullett SJ, Hinkle DA (2011) DJ-1 deficiency in astrocytes selectively enhances mitochondrial Complex I inhibitor-induced neurotoxicity. J Neurochem 117: 375–387.
41. Gonzalez-Polo R, Niso-Santano M, Moran JM, Ortiz-Ortiz MA, Bravo-San Pedro JM, et al. (2009) Silencing DJ-1 reveals its contribution in paraquat-induced autophagy. J Neurochem 109: 889–898.
42. Yamashita S, Mori A, Kimura E, Mita S, Maeda Y, et al. (2010) DJ-1 forms complexes with mutant SOD1 and ameliorates its toxicity. J Neurochem 113: 860–870.
43. Lev N, Ickowicz D, Barhum Y, Lev S, Melamed E, et al. (2009) DJ-1 protects against dopamine toxicity. J Neural Transm 116: 151–160.
44. Gu X, Neric NJ, Crabb JS, Crabb JW, Bhattacharya SK, et al. (2012) Age-related changes in the retinal pigment epithelium (RPE). PLoS One 7: e38673.
45. Chen M, Muckersie E, Robertson M, Fraczek M, Forrester JV, et al. (2008) Characterization of a spontaneous mouse retinal pigment epithelial cell line B6-RPE07. Invest Ophthalmol Vis Sci 49: 3699–3706.
46. Bonilha VL, Finnemann SC, Rodriguez-Boulan E (1999) Ezrin promotes morphogenesis of apical microvilli and basal infoldings in retinal pigment epithelium. J Cell Biol 147: 1533–1548.
47. Kinumi T, Kimata J, Taira T, Ariga H, Niki E (2004) Cysteine-106 of DJ-1 is the most sensitive cysteine residue to hydrogen peroxide-mediated oxidation in vivo in human umbilical vein endothelial cells. Biochem Biophys Res Commun 317: 722–728.
48. Wilson MA (2011) The role of cysteine oxidation in DJ-1 function and dysfunction. Antioxid Redox Signal 15: 111–122.
49. Ooe H, Iguchi-Ariga SM, Ariga H (2006) Establishment of specific antibodies that recognize C106-oxidized DJ-1. Neurosci Lett 404: 166–169.
50. Zhang L, Shimoji M, Thomas B, Moore DJ, Yu SW, et al. (2005) Mitochondrial localization of the Parkinson's disease related protein DJ-1: implications for pathogenesis. Hum Mol Genet 14: 2063–2073.
51. Ooe H, Taira T, Iguchi-Ariga SM, Ariga H (2005) Induction of reactive oxygen species by bisphenol A and abrogation of bisphenol A-induced cell injury by DJ-1. Toxicol Sci 88: 114–126.
52. Lev N, Ickowicz D, Melamed E, Offen D (2008) Oxidative insults induce DJ-1 upregulation and redistribution: implications for neuroprotection. Neurotoxicology 29: 397–405.
53. George S, Mok SS, Nurjono M, Ayton S, Finkelstein DI, et al. (2010) alpha-Synuclein transgenic mice reveal compensatory increases in Parkinson's disease-associated proteins DJ-1 and parkin and have enhanced alpha-synuclein and PINK1 levels after rotenone treatment. J Mol Neurosci 42: 243–254.
54. Inberg A, Linial M (2010) Protection of pancreatic beta-cells from various stress conditions is mediated by DJ-1. J Biol Chem 285: 25686–25698.
55. Knobbe CB, Revett TJ, Bai Y, Chow V, Jeon AH, et al. (2011) Choice of biological source material supersedes oxidative stress in its influence on DJ-1 in vivo interactions with Hsp90. J Proteome Res 10: 4388–4404.
56. Bitar MS, Liu C, Ziaei A, Chen Y, Schmedt T, et al. (2012) Decline in DJ-1 and decreased nuclear translocation of Nrf2 in Fuchs endothelial corneal dystrophy. Invest Ophthalmol Vis Sci 53: 5806–5813.
57. Joselin AP, Hewitt SJ, Callaghan SM, Kim RH, Chung YH, et al. (2012) ROS-dependent regulation of Parkin and DJ-1 localization during oxidative stress in neurons. Hum Mol Genet 21: 4888–4903.
58. Saito Y, Hamakubo T, Yoshida Y, Ogawa Y, Hara Y, et al. (2009) Preparation and application of monoclonal antibodies against oxidized DJ-1. Significant elevation of oxidized DJ-1 in erythrocytes of early-stage Parkinson disease patients. Neurosci Lett 465: 1–5.
59. Akazawa YO, Saito Y, Hamakubo T, Masuo Y, Yoshida Y, et al. (2010) Elevation of oxidized DJ-1 in the brain and erythrocytes of Parkinson disease model animals. Neurosci Lett 483: 201–205.
60. Kitamura Y, Watanabe S, Taguchi M, Takagi K, Kawata T, et al. (2011) Neuroprotective effect of a new DJ-1-binding compound against neurodegeneration in Parkinson's disease and stroke model rats. Mol Neurodegener 6: 48.
61. Hulleman JD, Mirzaei H, Guigard E, Taylor KL, Ray SS, et al. (2007) Destabilization of DJ-1 by familial substitution and oxidative modifications: implications for Parkinson's disease. Biochemistry 46: 5776–5789.
62. Blackinton J, Ahmad R, Miller DW, van der Brug MP, Canet-Aviles RM, et al. (2005) Effects of DJ-1 mutations and polymorphisms on protein stability and subcellular localization. Brain Res Mol Brain Res 134: 76–83.
63. Gorner K, Holtorf E, Waak J, Pham TT, Vogt-Weisenhorn DM, et al. (2007) Structural determinants of the C-terminal helix-kink-helix motif essential for protein stability and survival promoting activity of DJ-1. J Biol Chem 282: 13680–13691.
64. Lin J, Prahlad J, Wilson MA (2012) Conservation of oxidative protein stabilization in an insect homologue of parkinsonism-associated protein DJ-1. Biochemistry 51: 3799–3807.
65. Blackinton J, Lakshminarasimhan M, Thomas KJ, Ahmad R, Greggio E, et al. (2009) Formation of a stabilized cysteine sulfinic acid is critical for the mitochondrial function of the parkinsonism protein DJ-1. J Biol Chem 284: 6476–6485.
66. Kim SJ, Park YJ, Oh YJ (2012) Proteomic analysis reveals a protective role for DJ-1 during 6-hydroxydopamine-induced cell death. Biochem Biophys Res Commun 422: 8–14.
67. Jeong HJ, Kim DW, Kim MJ, Woo SJ, Kim HR, et al. (2012) Protective effects of transduced Tat-DJ-1 protein against oxidative stress and ischemic brain injury. Exp Mol Med 44: 586–593.
68. Robert G, Puissant A, Dufies M, Marchetti S, Jacquel A, et al. (2012) The caspase 6 derived N-terminal fragment of DJ-1 promotes apoptosis via increased ROS production. Cell Death Differ 19: 1769–1778.
69. Yu HH, Xu Q, Chen HP, Wang S, Huang XS, et al. (2013) Stable overexpression of DJ-1 protects H9c2 cells against oxidative stress under a hypoxia condition. Cell Biochem Funct.
70. Nowak JZ (2006) Age-related macular degeneration (AMD): pathogenesis and therapy. Pharmacol Rep 58: 353–363.
71. Ding X, Patel M, Chan CC (2009) Molecular pathology of age-related macular degeneration. Prog Retin Eye Res 28: 1–18.
72. Kinnunen K, Petrovski G, Moe MC, Berta A, Kaarniranta K (2012) Molecular mechanisms of retinal pigment epithelium damage and development of age-related macular degeneration. Acta Ophthalmol 90: 299–309.
73. Mohan R, Sivak J, Ashton P, Russo LA, Pham BQ, et al. (2000) Curcuminoids inhibit the angiogenic response stimulated by fibroblast growth factor-2, including expression of matrix metalloproteinase gelatinase B. J Biol Chem 275: 10405–10412.
74. Schutt F, Volcker HE, Dithmar S (2007) [N-acetylcysteine improves lysosomal function and enhances the degradation of photoreceptor outer segments in cultured RPE cells]. Klin Monbl Augenheilkd 224: 580–584.
75. Chan CM, Huang JH, Lin HH, Chiang HS, Chen BH, et al. (2008) Protective effects of (−)-epigallocatechin gallate on UVA-induced damage in ARPE19 cells. Mol Vis 14: 2528–2534.
76. Cao GF, Liu Y, Yang W, Wan J, Yao J, et al. (2011) Rapamycin sensitive mTOR activation mediates nerve growth factor (NGF) induced cell migration and pro-survival effects against hydrogen peroxide in retinal pigment epithelial cells. Biochem Biophys Res Commun 414: 499–505.
77. Chong EW, Wong TY, Kreis AJ, Simpson JA, Guymer RH (2007) Dietary antioxidants and primary prevention of age related macular degeneration: systematic review and meta-analysis. BMJ 335: 755.
78. Parisi V, Tedeschi M, Gallinaro G, Varano M, Saviano S, et al. (2008) Carotenoids and antioxidants in age-related maculopathy italian study: multifocal electroretinogram modifications after 1 year. Ophthalmology 115: 324–333 e322.
79. Bandopadhyay R, Kingsbury AE, Cookson MR, Reid AR, Evans IM, et al. (2004) The expression of DJ-1 (PARK7) in normal human CNS and idiopathic Parkinson's disease. Brain 127: 420–430.
80. Jarrett SG, Lin H, Godley BF, Boulton ME (2008) Mitochondrial DNA damage and its potential role in retinal degeneration. Prog Retin Eye Res 27: 596–607.
81. Turrens JF (2003) Mitochondrial formation of reactive oxygen species. J Physiol 552: 335–344.
82. Shabalina IG, Nedergaard J (2011) Mitochondrial ('mild') uncoupling and ROS production: physiologically relevant or not? Biochem Soc Trans 39: 1305–1309.
83. Santos JH, Hunakova L, Chen Y, Bortner C, Van Houten B (2003) Cell sorting experiments link persistent mitochondrial DNA damage with loss of

mitochondrial membrane potential and apoptotic cell death. J Biol Chem 278: 1728–1734.
84. Feher J, Kovacs I, Artico M, Cavallotti C, Papale A, et al. (2006) Mitochondrial alterations of retinal pigment epithelium in age-related macular degeneration. Neurobiol Aging 27: 983–993.
85. Nordgaard CL, Berg KM, Kapphahn RJ, Reilly C, Feng X, et al. (2006) Proteomics of the retinal pigment epithelium reveals altered protein expression at progressive stages of age-related macular degeneration. Invest Ophthalmol Vis Sci 47: 815–822.
86. Nordgaard CL, Karunadharma PP, Feng X, Olsen TW, Ferrington DA (2008) Mitochondrial proteomics of the retinal pigment epithelium at progressive stages of age-related macular degeneration. Invest Ophthalmol Vis Sci 49: 2848–2855.
87. SanGiovanni JP, Arking DE, Iyengar SK, Elashoff M, Clemons TE, et al. (2009) Mitochondrial DNA variants of respiratory complex I that uniquely characterize haplogroup T2 are associated with increased risk of age-related macular degeneration. PLoS One 4: e5508.
88. Kenney MC, Atilano SR, Boyer D, Chwa M, Chak G, et al. (2010) Characterization of retinal and blood mitochondrial DNA from age-related macular degeneration patients. Invest Ophthalmol Vis Sci 51: 4289–4297.
89. Mueller EE, Schaier E, Brunner SM, Eder W, Mayr JA, et al. (2012) Mitochondrial haplogroups and control region polymorphisms in age-related macular degeneration: a case-control study. PLoS One 7: e30874.
90. Stenirri S, Santambrogio P, Setaccioli M, Erba BG, Pia Manitto M, et al. (2012) Study of FTMT and ABCA4 genes in a patient affected by age-related macular degeneration: identification and analysis of new mutations. Clin Chem Lab Med 50: 1021–1029.
91. Blasiak J, Glowacki S, Kauppinen A, Kaarniranta K (2013) Mitochondrial and nuclear DNA damage and repair in age-related macular degeneration. Int J Mol Sci 14: 2996–3010.
92. Kenney MC, Hertzog D, Chak G, Atilano SR, Khatibi N, et al. (2013) Mitochondrial DNA haplogroups confer differences in risk for age-related macular degeneration: a case control study. BMC Med Genet 14: 4.
93. Crabb JW, Miyagi M, Gu X, Shadrach K, West KA, et al. (2002) Drusen proteome analysis: an approach to the etiology of age-related macular degeneration. Proc Natl Acad Sci U S A 99: 14682–14687.
94. Bando H, Shadrach KG, Rayborn ME, Crabb JW, Hollyfield JG (2007) Clathrin and adaptin accumulation in drusen, Bruch's membrane and choroid in AMD and non-AMD donor eyes. Exp Eye Res 84: 135–142.
95. Hageman GS, Mullins RF, Russell SR, Johnson LV, Anderson DH (1999) Vitronectin is a constituent of ocular drusen and the vitronectin gene is expressed in human retinal pigmented epithelial cells. FASEB J 13: 477–484.
96. Mullins RF, Russell SR, Anderson DH, Hageman GS (2000) Drusen associated with aging and age-related macular degeneration contain proteins common to extracellular deposits associated with atherosclerosis, elastosis, amyloidosis, and dense deposit disease. FASEB J 14: 835–846.
97. Anderson DH, Ozaki S, Nealon M, Neitz J, Mullins RF, et al. (2001) Local cellular sources of apolipoprotein E in the human retina and retinal pigmented epithelium: implications for the process of drusen formation. Am J Ophthalmol 131: 767–781.
98. Malek G, Li CM, Guidry C, Medeiros NE, Curcio CA (2003) Apolipoprotein B in cholesterol-containing drusen and basal deposits of human eyes with age-related maculopathy. Am J Pathol 162: 413–425.
99. Shimwell NJ, Ward DG, Mohri Y, Mohri T, Pallan L, et al. (2012) Macrophage migration inhibitory factor and DJ-1 in gastric cancer: differences between high-incidence and low-incidence areas. Br J Cancer 107: 1595–1601.
100. Lisitskaia KV, Eremina LS, Ivanov AV, Kovaleva MA, Okhrits VE, et al. (2011) [Study of Dj-1 protein in tissue specimens, cultured cells and serum of prostate cancer patients]. Biomed Khim 57: 392–401.
101. Chen Y, Kang M, Lu W, Guo Q, Zhang B, et al. (2012) DJ-1, a novel biomarker and a selected target gene for overcoming chemoresistance in pancreatic cancer. J Cancer Res Clin Oncol 138: 1463–1474.
102. Zeng HZ, Qu YQ, Zhang WJ, Xiu B, Deng AM, et al. (2011) Proteomic Analysis Identified DJ-1 as a Cisplatin Resistant Marker in Non-Small Cell Lung Cancer. Int J Mol Sci 12: 3489–3499.
103. Pardo M, Garcia A, Thomas B, Pineiro A, Akoulitchev A, et al. (2006) The characterization of the invasion phenotype of uveal melanoma tumour cells shows the presence of MUC18 and HMG-1 metastasis markers and leads to the identification of DJ-1 as a potential serum biomarker. Int J Cancer 119: 1014–1022.
104. Oda M, Makita M, Iwaya K, Akiyama F, Kohno N, et al. (2012) High levels of DJ-1 protein in nipple fluid of patients with breast cancer. Cancer Sci 103: 1172–1176.
105. Hong Z, Shi M, Chung KA, Quinn JF, Peskind ER, et al. (2010) DJ-1 and alpha-synuclein in human cerebrospinal fluid as biomarkers of Parkinson's disease. Brain 133: 713–726.
106. Shi M, Furay AR, Sossi V, Aasly JO, Armaly J, et al. (2012) DJ-1 and alphaSYN in LRRK2 CSF do not correlate with striatal dopaminergic function. Neurobiol Aging 33: 836 e835–837.
107. Salvesen L, Bech S, Lokkegaard A, Hjermind LE, Nielsen JE, et al. (2012) The DJ-1 concentration in cerebrospinal fluid does not differentiate among Parkinsonian syndromes. Parkinsonism Relat Disord 18: 899–901.
108. Abdalla MA, Haj-Ahmad Y (2012) Promising Urinary Protein Biomarkers for the Early Detection of Hepatocellular Carcinoma among High-Risk Hepatitis C Virus Egyptian Patients. J Cancer 3: 390–403.
109. Davis AA, Bernstein PS, Bok D, Turner J, Nachtigal M, et al. (1995) A human retinal pigment epithelial cell line that retains epithelial characteristics after prolonged culture. Invest Ophthalmol Vis Sci 36: 955–964.
110. Nandrot EF, Kim Y, Brodie SE, Huang X, Sheppard D, et al. (2004) Loss of synchronized retinal phagocytosis and age-related blindness in mice lacking alphavbeta5 integrin. J Exp Med 200: 1539–1545.
111. Bonilha VL, Shadrach KG, Rayborn ME, Li Y, Pauer GJ, et al. (2013) Retinal deimination and PAD2 levels in retinas from donors with age-related macular degeneration (AMD). Exp Eye Res 111C: 71–78.
